# Malnutrition and female political representation in India

**DOI:** 10.1371/journal.pone.0342588

**Published:** 2026-03-18

**Authors:** Parul Tyagi, Philippe LeMay-Boucher

**Affiliations:** Department of Economics, Heriot-Watt University, Edinburgh, United Kingdom; University of Pretoria, SOUTH AFRICA

## Abstract

Malnutrition casts a negative shadow over the course of the lives of a fifth of children under the age of five who are stunted worldwide. Despite two decades of growth, India has one of the highest wasting and stunting rates. Lack of political awareness and poor governance, arguably related to an acute underrepresentation of women in politics in India may explain why not enough is committed to this matter. Increasing women’s political agency in elected bodies has brought several documented benefits yet little is known about its impact on children’s nutrition outcomes. Our analysis shows that increasing women’s political representation in India’s state legislatures could reduce child malnutrition. We use a large cross-sectional district representative household survey collected between 2002−4 through the second round of the District Level Household Survey. It is merged with detailed state legislative assembly election data we collected. These detail who contested seats in each constituency and the gender composition of elected representatives in India’s 17 largest states between 1993 and 2004. We find that a ten-percentage point increase in women’s representation leads to a three to five percentage point reduction in the likelihood of a child being underweight. Malnutrition is a multi-faceted issue with different underlying causes, and our work explores some of these. We document that greater female political representation creates productive ways to enhance public health by bringing improvements in households’ access to basic amenities and to prenatal, birth and postnatal care among mothers.

## Introduction

Worldwide 22% of children under the age of five are stunted according to [1]. 45.4 million children under five suffer from wasting (moderate and severe), with 12.1 million in Africa and 31.9 million in Asia. Evidence suggests that undernutrition casts a negative shadow over an individual’s course of life. On average, undernourished children are less healthy, develop weaker cognitive skills, and ultimately experience lower productivity and earnings [2, 3]. Malnutrition is responsible for approximately 45% of deaths for children under the age of five and increases children’s vulnerability to common childhood illness such as malaria, pneumonia, and diarrhoea [4].

Western countries that moved up the income ladder saw improvements in their population’s height rates [5]. However, in Asia and Africa notably, the relationship between national income and population heights is inconsistent and unreliable [6]. India, the second most populous country and one of the demographically youngest, provides a striking example of this disconnect. Between 1995 and 2019, India’s GDP grew annually between 3% and 9% (at an average rate of 6.55%). Despite improvements in food security, education, life expectancy, and maternal mortality [3], India has one of the highest wasting (17.3%, 2017) and stunting rates (30.9% in 2020 down from 41.7% in 2012) in the world. This represents a quarter of all stunted children globally [1]. [7] suggests that 68 percent of children-under-five mortality in India is due to malnutrition.

What explains this disconnect? A strand of the literature argues that poverty alone is not a persuasive explanation for the high prevalence of child malnutrition and mortality, as most could be prevented with low-cost interventions [8,9]. Some evidence points to a lack of political awareness around malnutrition [10,11], issues of poor governance and lack of interventions specifically related to nutrition [11,12]. In some cases, governments do not commit or prioritise enough provision of food, sanitation, water, care, and health services.

Another strand of literature shows that women’s empowerment (or autonomy) within their households positively impact their own and children’s nutrition [13–16]. Studies also show that at the individual level women invest more than men in children’s education, childcare, and other family-benefiting expenditure [17–20]. Furthermore, men’s and women’s respective financial and time contributions to their households is likely to impact their concerns over public resources. Yet limited research exists on how women’s political representation might influence child nutrition outcomes.

Evidence from developing nations that women leadership improves public health outcomes is limited. The 73^rd^ constitutional Indian amendment act in 1993 required the states to devolve more powers over expenditures to local village councils (Gram Panchayats) and allocate one-third of all positions of chief (Pradhan) to women. Subsequent elections delivered that threshold in most major states. [21] surveys investments in local public goods in a sample of villages and compare them for reserved and unreserved village councils. Their findings indicate that reservation affects policy choices in ways that seem to better reflect women’s preferences. They attribute their results to the reserved status of those village councils given that they were randomly selected to be reserved for women. Likewise, council reserved for female leaders increases the likelihood of learning, nutrition outcomes for children, while also improving employment opportunities and aspirations among females and decreasing crime rate against women [22–25]. Related studies focus on women political representation in Indian state legislative assembly, where, contrary to Pradhan (village head) positions, reservation rates have yet to be applied. [26], focusing on the years 1967–1999, finds that an increase in women’s representation at the district level leads to a significant reduction in neonatal mortality. [27] reports that such increase also improves females’ (aged 15–24) labour market outcomes.

Our retrospective study is distinct in that it examines the impact of women representation in elected bodies on undernutrition outcome for children aged under five at the state level. Given the important role that the states of India play on public health policies our work addresses a relevant research question. Additionally, our retrospective analysis is based on a different time-period and employs different data: a large household survey which has a better suited representativity at the district level.

Our work provides additional pertinence to the claim that it is plausible that women’s political representation at state level is an underutilized tool for addressing health issues in developing countries. Members of a state legislative assembly (MLA) are elected at the constituency level and are responsible, among other things, for local amenities. MLAs can direct funds to help public health infrastructure at the district level which can bring direct and indirect effects to their constituencies. MLAs can also influence public services delivery mechanism though their close interactions with village and district administrations [28, 29]. Districts often act as an important level of administration between the state and the village level local governments. Given India’s poor track record in relation to stunting and wasting [1], one can argue that its authorities have either promoted inefficient policies or given this issue low priority. Increasing women political representation could change this. It could come from their influence on policies related to underlying causes of malnutrition. [30] provides evidence that, among others, safe hygiene and sanitation practices, availability of health care facilities and healthy environmental factors improve child nutrition outcomes. With our data, we can investigate, in Section ‘Discussion on channels’, the influence of female political representation on a range of related indicators: 1) antenatal and delivery care; 2) postnatal care, child feeding practises, and caregiver awareness and 3) household access to basic amenities. To the best of our knowledge, there is little evidence of such causal impact on children’s anthropometry outcomes based on actual electoral outcome rather than reservation policies.

To assess this, we use a large cross-sectional district representative household survey collected between 2002−04 through the second round of the District Level Household Survey (DLHS-2). This survey round is the only one to include extensive data on children’s anthropometric measurements. Our dependent variables related to nutrition outcome are thus based on a district representative survey avoiding us using national representative survey at the state level. Alongside this, we collected detailed state legislative assembly election data on electoral candidates in each constituency in the 17 largest states of India between 1993 and 2004. The states included in the study are the largest states of India, which account, for that period for more than 97 percent of India population. They are AP, Assam, Bihar, Gujrat, Haryana, HP, Karnataka, Kerala, MP, Maharashtra, Orissa, Punjab, Rajasthan, TM, Uttar Pradesh, Jammu and Kashmir, and West Bengal. Three new states —Chhattisgarh, Jharkhand, and Uttarakhand – were carved out in 2001 from MP, Bihar and Uttar Pradesh, respectively. Therefore, newly created states were not included in the analysis. We map the DLHS-2 survey data with the electoral data based on district identification of the households. Our final sample includes the 17 largest states and covers 361 districts that remain intact over time between the 1991 and 2001 census. These constituency level data, detailing the gender composition of elected representatives, are aggregated at the district level. Using district level data is made necessary by the fact that DHLS-2 households’ geographical location is only given at that level. In Section S1 of the Supporting Information (S1 Appendix), we provide a detailed explanation of why we use this wave of the DLHS and why we use DLHS data over other alternative surveys such as NFHS and IHDS.

The main challenge in estimating the causal effect of female politicians on children’s nutrition outcome is the presence of unobserved factors such as electoral preferences. It is possible that districts with less stringent gender norms may prefer to elect female politicians. Therefore, to address the endogeneity of electoral preferences, our identification strategy uses the proportion of seats won by women in close elections between women and men candidates, as an exogenous instrument for proportion of seats won by women in a district. The idea is that gender identity of the winner in close man-woman elections is assumed to be quasi-random as there is no clear indication of an electoral preference for either male or female politicians. Given that election data is aggregated at district level, we implement a design similar to a fuzzy RD.

Overall, our findings suggest that politicians’ gender significantly matters for children’s nutrition outcomes: whether a child is underweight (weight-for-age z-score < −2), severely underweight (weight-for-age z-score < −3), and their continuous weight-for-age z-score. The effects of an increase in female political representation in reducing malnutrition are strong and statistically significant.

The remainder of this article is organized as follows. Section ’Background’ describes the institutional context and explains how women’s political representation can affect nutrition. The next provides details on the data used and presents descriptive statistics. We then outline our empirical strategy, detail and discuss our results, followed by robustness checks and a conclusion.

## Background

### Women in politics

139 countries have now adopted constitutional, electoral or political party gender quotas at either national or sub-national levels (taken from the Gender Quotas Database (International Institute for Democracy and Electoral Assistance: idea.int), retrieved April 2024). Since the early 90s, there has been a significant increase in share of women in parliaments worldwide, standing at 21% in 2020 [31]. Yet, there remains a considerable under underrepresentation of women in politics in India. The World Economic Forum ranks it 118^th^ (out of 143 countries) for its share of women in parliament and 126^th^ for its share of women in ministerial position [32].

The literature based on the ‘citizen candidate model’ suggests that the identity of politician will matter for policy purposes because political representation of a group increases its influence in policy making [33, 34]. In the absence of complete policy commitment, the gender of a politician matters for policy outcomes. Studies from developed countries suggests that the gender of legislator makes for different policy choices. Using data on local council elections in Bavaria, [35] show that an increase in female councillors expands significantly public childcare. [36] based on the analysis of Swedish municipalities, concludes that rising share of women council seats results in higher spending related to child and education related to elderly. [37] finds that women US legislator prefers to spend more on family assistance services and childcare. Further, there is also evidence that women politicians are more liberal than men, and more likely to express concerns linked to social issues [38, 39].

In India, the 73rd constitutional amendment act in 1993 reserved one-third of seats for women in village councils. [21] study the effect of women’s headship at local council on village level spending patterns in West Bengal. They estimate a causal effect by using the fact that one third of seats in local council pradhan (village headship) was randomly assigned to female. The finding shows that female leaders spend more on public services related to women’s need. Likewise, council seats reserved for female leader improve learning, and early child development in Andhra Pradesh [22] and aspirational outcomes for females in West Bengal [24]. [40] documents that areas that are more exposed to female leaders in village councils allocate higher share of public work employment to constituent women. Evidence also suggests that women entrepreneur in India increased with the adoption of women political reservations [23]. These findings tend to indicate that female politicians are relatively more attentive to women’s needs than male politicians.

These studies identify the impact of village council headship rather than women representation. Our work differs with that regard and use women’s political representation in state legislatures assemblies rather than village council’s headship. Closest to our work are the following two papers who studied the impact of women’s representation in elected bodies on health and education outcomes. [29] shows that increasing the share of women representation in politics results in higher education outcomes only in urban areas. [26] concludes that there is an impact of women representation on mortality rates (negative) and the use of health facilities (positive). They also find that female legislators invest more in village level health facilities than men legislators. By extension a larger female representation may help in establishing successful programmes that target the underlying causes of malnutrition. These depend on multisectoral interventions from various areas such as agriculture, social-safety net, early childhood development, and schooling [30]. Female politicians may be more inclined to promote safe hygiene and sanitation practices, health care facilities availability and healthy environmental factors: all proven factor in improving child nutrition outcomes [30].

[12, 41] bring additional relevance to our research question. They suggest there is a need to focus on how leadership, political commitment, and accountability provide a supportive environment for malnutrition reduction. The immediate and underlying causes of child undernutrition often depend on the larger issues of poor governance and lack of attention towards policies related to nutrition programmes [11]. Given the consistently high levels of malnutrition we observe in India, we can surmise that decisions made by the country’s predominately male policymaking bodies may not reflect the policies prioritising solutions to its causes. It is worth investigating empirically whether an increase in women’s representation in politics reduces malnutrition.

### India’s political structure

India has a three-tier federal structure which is generally defined as central, state, and local level of representation. India comprises 28 states, and 9 union territories. All states and union territories are sub-divided into districts and smaller administrative divisions. The fundamental constitutional power comes directly from an elected parliamentary style government at the national and state level. At the national level, India follows a provision of bicameral legislature that consists of the President of India, and two houses of representatives. The upper house known as *Rajya Sabha* (Council of States), and the lower house known as *Lok Sabha* (House of the People). The states and union territories have their own legislative assembly, and Chief Ministers lead the executive. The state and union governments are formed every five years through election. A member of the legislative assembly (MLA) is elected based on a first-past-the-post system in a single-member-constituency by the electorate. State legislative assemblies vary in size according to state populations. Their main purpose is to legislate in areas in which the national parliament is not allowed to. Some of these include public order, agriculture, irrigation, public health, local government, and pilgrimage. There are some areas where both parliament and states jointly make laws (education, marriage, environment, etc.). Any constitutional amendment must be agreed by both houses of parliament and ratified by at least half of the state legislatures.

Following decentralization, the central government agreed that local government (for example Zila Parishad, village pradhan) should be given constitutional status. The objective was to strengthen local administrative units for economic development where policy decisions can be implemented at the micro level. As mentioned above, the 73^rd^ and 74^th^ constitutional amendments gave recognition and power to local governments. More authority was delegated to district and local governments and introduced quotas for women at village levels. Specifically, one third of seats in every village council and, by random allocation, one third of posts of head of a village council (*Pradhan*) have been reserved for women. The introduction of village level reservation has been considered as a success for women’s movement. In September 1996, the Government of India introduced a bill in the parliament to reserve one third of the seats for women in the central government (*Lok Sabha*) and state legislative assemblies. This bill was recently passed (September 2023) by the Lower House *(Lok Sabha)* and the Upper House *(Rajya Sabha)* with no clear indication as to how soon it will be implemented (most certainty not before 2029, see https://www.ndtv.com/india-news/womens-reservation-bill-ndtv-explains-how-soon-can-womens-quota-bill-come-into-effect-4408298). Those who favour such reservation policies argue that increasing women’s political representation at the higher level will ensure a better representation of their needs. Our work uses female political representation based on state legislative elections results for which no reservation has been implement yet.

### Data

#### Election data.

Information on the proportion of state seats won by women politician were extracted from the numerous Election Commission of India reports on the State Legislative elections for the 1993–2004 period. The Election Commission is an independent agency established by the Constitution of India to conduct and control elections in the country. State elections in India were not concurrent for the focus period of this study (1993–2004). We do not control for the different timing of elections (month or quarter within a given year) across states and neither does the related literature. It is the authoritative source on election data. These reports contain information on constituency level data for each state elections. On winner and runner-up candidates, their gender and party affiliation and electoral turnout for every state legislative election in India. Data on candidates’ age, education level, assets and other metrics only became available after 2004. The sample we use contains information on 3367 constituencies for each election year in the 17 largest state legislative elections. The number of constituencies in each district ranges from 1 to 37 with a mean of seven.

Women political representation in India is low. As shown in Table 1, across districts, the average proportion of seats per district won by women politician is 0.062 (standard deviation of 0.09). The distribution of this proportion is heavily skewed towards zero with a median of zero and the 75^th^ and 95^th^ percentiles at 0.11 and 0.22 respectively. On average, 40.8 percent of districts had at least one female politician. Amongst the districts with at least one female politician, 15.1 percent of seats were held by a female politician.

**Table 1:** Descriptive statistics for election data at the state level (17 states; 1993-2004).

Election variables (number of obs = 2130)	Mean	SD	Min	Max
Proportion of seats in a district won by female politician	0.062	0.09	0	0.6
Proportion of seats in a district that had close elections (margin of victory<3.5%) between female and male candidates*	0.022	0.06	0	0.33
Proportion of seats in a district won by female politician in close election (margin of victory<3.5%) against male politician*	0.008	0.03	0	0.2
Proportion of districts that had at-least one election between female and male candidates	0.588	0.49	0	1
Proportion of districts that had at-least one female politician	0.408	0.49	0	1
Proportion of seats in a district won by female politician that had at-least one female politician (number of obs = 868)	0.151	0.09	0.04	0.6
Proportion of seats reserved for SC/ST candidates	0.229	0.19	0	1
Proportion of seats won by SC/ST women	0.021	0.06	0	0.6
Proportion of seats in a district that had close elections (margin of victory<2%) between female and male candidates	0.014	0.05	0	0.33
Proportion of seats in a district won by female politician in close election (margin of victory<2%) against male politician	0.006	0.03	0	0.2
Proportion of seats in a district that had close elections (margin of victory<2.5%) between female and male candidates	0.02	0.05	0	0.33
Proportion of seats in a district won by female politician in close election (margin of victory<2.5%) against male politician	0.007	0.03	0	0.2
Proportion of seats in a district that had close elections (margin of victory<4%) between female and male candidates	0.024	0.06	0	0.4
Proportion of seats in a district won by female politician in close election (margin of victory<4%) against male politician	0.009	0.03	0	0.2
Proportion of seats in a district that had close elections (margin of victory<4.5%) between female and male candidates	0.027	0.06	0	0.4
Proportion of seats in a district won by female politician in close election (margin of victory<4.5%) against male politician	0.010	0.03	0	0.2
Proportion of seats in a district that had close elections (margin of victory<5%) between female and male candidates	0.029	0.07	0	0.4
Proportion of seats in a district won by female politician in close election (margin of victory<5%) against male politician	0.012	0.04	0	0.2

Note: *close elections are defined as elections where the vote difference between winner and runner-up candidate is less than 3.5%.

Table 1 shows that around 58 percent of the districts had at-least one election in which a male candidate faced a female candidate. 2.2 percent of seats had close elections between male and female politicians. Of these, 36% were won by a female (that represents the 0.8 percent on the third line). We define close elections as elections where the vote difference between winner and runner-up candidate is less than 3.5 percent (Section ‘Empirical strategy’ further justifies this threshold). In our sample, 22.9 percent of seats were held by a scheduled caste or scheduled tribe politician. Amongst these seats only 2.1 percent were won by a female politician. Female representation at the national level have been low for the period covered by our work. The proportion of seats won by women at the State level elections oscillate around 4.5 percent between 1993 and 1997 and then follows a strong upward trend up to around 7 percent in 2002.

### Nutrition data

We use nutrition data from the second round of the District Level Household Survey (DLHS-2) which were collected between 2002 and 2004 [42]. DLHS-2 survey provides cross-sectional information on anthropometry measurement of weight for children aged 0−71 months. The DLHS was used by the Government of India for monitoring 1) people’s perceptions of the quality of health services; 2) the utilization of health facilities provided both by the government and private parties; and 3) the progress made with respect to the Reproductive and Child Health (RCH) programme. This required district level data. Round two of the survey was completed between 2002−04 in 593 districts. The district level household survey covered a representative sample of about 1,000 households in each district. All married women aged 15−44 in the sample were interviewed. More details on this survey, sampling and data can be found here: http://rchiips.org/pdf/rch2/National_Report_RCH-II.pdf. The first round (DLHS-1: 1998−1999) and third round (DLHS-3: 2007−2008) of this large-scale survey cannot be used for the purpose of this study due to them lacking anthropometric data. DLHS-2 covers the whole of India and is nationally representative, with 593 districts across 36 states and union territories. In Section S1 of the Supporting Information (S1 Appendix), we provide a detailed explanation of why we use this wave of the DLHS, and not a more recent one; and why we use DLHS data over other alternative surveys such as NFHS and IHDS.

For children under five, one widely used measure for assessing nutrition status is weight-for-age (WAZ) which represents the body mass relative to age [43]. Another widely used index based on the height of the child (height-for-age) could not be generated for this study as children’s height was not collected by the DLHS-2. WAZ is used to measure chronic and acute malnutrition among children due to early childhood exposure to unfavourable conditions. These normally include illness, infections, nutritional deficiencies, and poor diets. Children’s weight is used to construct weight-for-age z-scores, which were estimated using 2006 WHO child growth standards reference by gender [43]. Children’s anthropometry data based on weight, age, and sex were converted into continuous WAZ z-scores variable using the STATA zscore06 command. Observations falling above plus or minus five standard deviations of the sample are potentially due to measurement error or incorrect age reporting and were excluded from the analysis [43]. Following WHO guidelines, we use z-score cut-off of less than two standard deviations to identify children as underweight. Underweight measures an acute and chronic form of nutritional deprivation [43]. The binary variable for underweight thus takes value one if a child’s weight-for-age z-scores is smaller than −2 and zero otherwise. In addition, we look at severely underweight children who are classified as children with weight-for age z score (WAZ) of less than −3 (as per WHO guidelines). These children have very high risk of death [44].

As shown in Table 2, the total number of children under five in our sample is 122,926: 48 percent girls and 52 percent boys. Estimates show that around 41 and 45 percent of children under two years and children under five years are underweight respectively. Around 20 percent of children under five are severely underweight.

**Table 2:** Descriptive statistics at the individual level (DHLS cross section data collected between 2002−04).

	Mean	SD
***Outcome variables (as per WHO 2006 guidelines or otherwise stated)***		
Children underweight (0–60 months)	0.45	0.50
Children severely underweight (0–60 months)	0.20	0.40
Children underweight (0–24 months)^a^	0.41	0.49
Children severely underweight (0–24 months)^a^	0.20	0.40
Children weight-for-age z-score (0–60 months)	−1.78	1.45
Children weight-for-age z-score (0–24 months)^a^	−1.57	1.64
Children weight-for-age z-score (0–60 months) (as per NCHS)*	−1.83	1.40
Children weight-for-age z-score (0–24 months) (as per NCHS)*^a^	−1.54	1.61
***Controls: Individual, household and village level variables DLHS 2***		
Child is a girl	0.48	0.50
Mother’s education (in years)	4.01	4.81
Mother’s age at child’s birth	23.26	5.06
Live in rural area	0.73	0.45
Proportion of villages in a district with Health center	0.47	0.18
Proportion of villages in a district with government health center	0.50	0.19
*Household caste*		
Scheduled caste (SC)	0.21	0.41
Scheduled tribe (ST)	0.09	0.29
All other caste	0.70	0.46
*Household Religion*		
Hindu	0.81	0.39
Muslims	0.14	0.34
All other	0.05	0.22
*Household standard of living index*		
High	0.19	0.39
Medium	0.31	0.46
Low	0.49	0.49
***Controls: District level controls (Census)***		
Proportion of urban population in a district	0.23	0.16
Proportion of scheduled caste and schedule tribe population in a district	0.25	0.14
Proportion of female population in a district	0.48	0.01
Proportion of literacy rate in a district	0.61	0.13
**Observations**	**122,926**	

Note: *NCHS estimates for WAZ are based on DLHS estimates. ^a^ sample for these variables is 46349 obs.

DLHS-2 data also provides information on married women aged 15–44 years, who live with the households selected for the survey. The relevant women survey part covers information on fertility, primary healthcare, family planning, healthcare awareness, and utilization of health services such as childcare, antenatal and postnatal care during pregnancy. We make use of this information when analysing the potential channels through which female representation impacts malnutrition in Section ’Discussion on channels’.

Ethical considerations: This study is a secondary analysis of de-identified publicly available national databases. The authors accessed the data on March 2^nd^, 2020. All participants provided written informed consent to participate in the survey. For minors, written consent was obtained from parents or guardians. The DLHS-2 received ethics approval from the International Institute for Population Science’s Ethical Review Board and the Ministry of Health and Family Welfare, Government of India.

### Sample selection and merging nutritional and electoral data

The DLHS-2 data we use is a cross-sectional household survey designed to be representative at the district level. Households’ geographical location is given at district level only. The election data are at the constituency level, and most districts consist of several constituencies (ranging from 1 to 37; mean of 7). Merging these data requires aggregating the electoral data from constituency level to district level by averaging. For example, if there are 10 constituencies in a given district and female politicians won 4 seats; the proportion of seats won by female in that district is 0.4.

We use the *State Elections in India*, a publication of the Election Commission which lists constituencies included in each election year together with constituency delimitation order. This order links all electoral constituencies to their parent districts. The government of India’s 1976 delimitation order was replaced with a new one in 2002 which was first implemented in 2008. Thus, the electoral constituency boundaries remain unchanged between 1976 and 2007. However, some districts were either dissolved, or divided, or newly formed between 1976 and 2007. In the case of newly formed district, the mapping involves a transfer of constituencies from one district to another. Any source of mapping error at district level is thus ruled out. In our sample, all state assembly elections held between 1991 and 2004 are considered. The aggregation of constituency data to the district level is based on the 1991 census organised by the Government of India. For our analysis we use the 1991 census definition and only use districts that remained intact over time in 17 states in the 1991 and 2001 census. The 1991 census lists 411 districts in the 17 biggest states of India covering 97 percent of the population in India. We have dropped from the analysis newly created or deleted districts and some that became part of new states after division (e.g.,: Bihar split into Jharkhand in November 2000). The idea is to use districts in these 17 states that remained intact over time in the 1991 and 2001 censuses. The final sample we use consists of 361 districts in these 17 states.

We use DLHS-2 anthropometry data for children aged 0–5 when surveyed. This means we have cohorts of children being born for every single year between 1997 and 2004. We consider each children’s cohorts (birth year) as time-period *t*. The merging of the DLHS-2 and the election data is based on a child’s birth year. For a 5-year-old child surveyed in 2002 we merge the information on that child from the DLHS-2 using district identification of the household with the corresponding election data on women’s political representation at the district level for the year 1997. Our overall sample consists of 122,926 children aged 0–5 years born between 1997 and 2004. Further below, we consider as a robustness check a moving average of three years of the proportion of seats won by female politicians prior and including when a child was born.

### Empirical strategy

We aim to estimate the causal effect of women’s political representation on children’s nutritional outcome with the following model:


$${Yi}_{dt}\;=\;\alpha \;+\;\beta Womenpolitician_{dt}\;+\;{\varepsilon i}_{dt}$$ (1)

where ${Yi}_{dt}$ is the nutrition outcome (WAZ) for children *i*, living in district *d*, and born in cohort *t*. Using district *d* (and not constituency) is required as households’ geographical location is known at district level only. ${Womenpolitician}_{dt}$ is the proportion of constituency seats in a district *d* held by women politicians in the year of the childbirth. β is the main parameter of interest. To avoid any confusion, our estimations are based on the DLHS-2 cross-sectional data. The index *t* above in model 1 is used to match the birth of a child with the corresponding female political representation in the same year *t*. Our analysis is not based on panel nor pooled cross-sectional data.

When employing OLS to estimate β, the main challenge to identification strategy is the potential presence of omitted variables. One such widely acknowledged in the relevant literature is voters’ preferences, which likely drives both women politician representation and child nutrition outcome. District level fixed effects can control for time invariant effect across districts. However, it is difficult to rule out the possibility that the omitted variable may be district specific and change over time such as voters’ preferences. For example, districts with less stringent cultural norms could prefer and elect more female politicians. It is also possible that children with good nutrition may live in a progressive or liberal district with less restrictive gender norms and may thus prefer women politicians. Therefore, OLS estimates may not capture exclusively the causal impact of women politician on nutrition outcomes for children.

### Identification

To tackle this potential problem our estimation strategy takes advantage of close elections between male and female candidates. We define close elections as those where the margin of victory is less than 3.5 percent of the vote share between the winner and runner-up candidates. Our identification strategy uses the proportion of seats won by women in close elections between women and men candidates, as an exogenous instrument for the proportion of seats won by women in a district. The argument supporting this approach is that the small margin of victory (or vote share difference) between candidates of opposite sex in an election is a quasi-random outcome. Conditional on electoral turnout and other characteristics on the day of election, if there exists an element of uncertainty regarding the final vote share, then a winner in a closely contested election is determined by chance. The election of a candidate in closely contested election indicates that the district level preferences of electorates are unlikely to be gender biased. The crucial assumption here is that the constituencies in which females win close elections against male candidates are similar to the constituencies in which males have a close win. The cutoff of 3.5% is sufficiently close to zero while allowing a sufficient number of close elections in our sample. Key papers in that literature [29] have adopted that cutoff while showing additional results with various cutoff victory margins. We perform the same exercise with alternative victory cutoffs ranging from 2 to 6% and find similar results (see Section ‘Alternative thresholds for close elections’).

While the outcome of close elections between male and female candidates is considered random, the occurrence of such an election in a constituency may not be random. It may depend on factors such as the total number of woman candidates contesting a particular constituency. Therefore, the exclusion condition is satisfied by controlling for the share of constituencies in a district that had close elections between a male and a female candidate. The econometric model also controls for the polynomials of vote margin for all elections in a district where the top two candidates are of opposite gender. The forcing variable of vote margin captures the probability of treatment and is included in the regression to control for any relationship between the nutrition outcome and vote margins. During the considered period of 1993–2004, the maximum number of such close elections is seven in any given district.

In a first-past-the-post electoral system, the probability of winning an election is a function of vote difference between the winner and runner up candidates. This function has a discontinuity at zero. As vote difference approaches zero, the constituencies in which a female won by a small margin can be considered similar to the constituencies in which a male won by a small margin [45,46]. At the constituency level, the outcome of a closely contested election can be used to implement sharp regression discontinuity (RD) design. Because our analysis is based on election results a fuzzy regression discontinuity is needed [47]. This allows us to measure a discontinuity in the probability of treatment assigned to capture the causal effect of female politicians. The relevant graphical plot of fuzzy-RD identification strategy is presented in S1 Fig (shown in Section S1 of S1 Appendix). The fuzzy-RD plot shows the proportion of seats in a district won by women politician against the vote margins of victory or defeat between man-woman elections. Following [48], data for victory margin between man-woman elections are aggregated into one percentage bin points and a lowess smoothing line is plotted on each side of the discontinuity. A positive vote margin indicates that the women won the election, and the negative vote margin implies defeat (or men won). In Figure A1, the sharp jump at the discontinuity threshold of zero suggests that female winning in a close election in a district increases the overall share of seats won by female politicians in a district by roughly 7 percentage points. Section S1 of S1 Appendix shows various additional tests supporting the validity of this identification strategy. First, we provide evidence supporting the randomness of the outcome of a close election (Section S1.1). Second, we show, along a set of characteristics, that the districts in which female candidates won in close elections against men are similar along observables to those in which male candidates won in close elections against women (S1.2). Third, we provide evidence that districts that had close elections between men and women are not systematically different from other districts in India (S1.3). Fourth, we show that pre-determined covariates do not display jumps at the discontinuity point (S1.4).

### Estimation model

Estimations are conducted using a two-stage least square (2SLS) IV model.

With the following second stage equation:


$$Y_{idt}=\phi_{d}+\;\theta_{t}+\;\beta {Womenpolitician}_{dt}+\;\delta {TotalClose}_{dt}+\;\sum_{j=\text{1}}^{N}\delta_{\text{1}j}E_{jdt}\times G(M_{jdt})+\;\sum_{j=\text{1}}^{N}\delta_{\text{2}j}E_{jdt}+\;\alpha X_{idt}+\;\omega H_{idt}+\varsigma D_{idt}+\;\varepsilon_{idt}$$ (2)

And the corresponding first stage equation:


$${Womenpolitician}_{dt}=\emptyset_{d}+\;\gamma_{t}+\;\delta {WomenClose}_{dt}+\;\tau {TotalClose}_{dt}$$

$$+\sum_{j=\text{1}}^{N}\gamma_{\text{1}j}E_{jdt}\times G(\;M_{jdt})+\;\sum_{j=\text{1}}^{N}\gamma_{\text{2}j}E_{jdt}+\alpha X_{idt}+\;\omega H_{idt}+\varsigma D_{idt}+\;\varepsilon_{idt}$$ (3)

In equation 2, $Y_{idt}$ is the outcome variable. We initially use two dummy variables: *underweight* takes value 1 if child *i* from cohort *t* (birth year) and born in district *d* has a WAZ smaller than minus two and zero otherwise. *Severely underweight* takes value 1 if child has a WAZ smaller than minus three and zero otherwise. The independent variable of interest ${\;Womenpolitician}_{dt}$ is the proportion of seats in a district won by female politicians in the year and district where child *i* was born. We use the exogeneous instrumental variable ${WomenClose}_{dt}$ in equation 3. This variable is the proportion of seats in a district won by a female politician in a close election against a male runner-up (with a threshold of 3.5 percent). Although the outcome of close election is considered random, the presence of close elections between male and female candidates may not be. We also control for ${TotalClose}_{dt}$ in equation 3 which is the proportion of constituencies in each district that had close elections between a male and female candidate (with a threshold of 3.5 percent). Considering fuzzy regression discontinuity, the forcing variable is$\;M_{jdt}$ which stands for the margin of victory or defeat (as a fraction of total vote) of each of the female candidates who contested against a man in the woman-man constituency election *j*, in a district *d*, for the year *t*. We control for first, second and third order polynomials. *G* is the function of these polynomials based on victory margins (*M*) for all the male and female elections (close or non-close) between winner and runner-up candidates. These margins also interact with the dummy variable$\;E_{jdt}\;$which takes value one if there was a female and a male for either winner or runner-up (or vice-versa) in constituency election *j* in the district *d* and time *t*; and zero otherwise.

The vector of control variables $X_{idt}$ lists characteristics of mothers and children: gender of child, age of child (in months), age of mother in years at the time of birth of child, and the mother’s education level in years completed. $H_{idt}\;$includes household level characteristics: caste, religion, if located in rural area, and socio-economic status index (SSE). Caste is represented by a series of dummy variables classifying whether a household belongs to scheduled caste (SC), scheduled tribe (ST), or non-scheduled status. A series of dummy variables is used to represent the religion of households: Muslim, Hindu, or any other. To control for socio-economic status, we use the DLHS estimate for the standard of living index (SSE). The SSE index measures the overall living standard of a given household. It captures several dimensions: household level amenities (such as pucca house, source of lighting fuel, source of drinking water, toilet facility) and ownership of durable goods. Based on these scores, SSE is divided into three categorised within the DLHS sample: low, medium, and high standard of living.

For district level controls, we use census data. They account for any district level time varying preferences of electorate which may vary across districts.$\;D_{idt}$ includes district-year varying controls such as urban population, SC/ST population, female population, and literacy rate. These variables are interpolated between census years to get values for each year when female politician was in power. For district level control variables, we have used the 1981, 1991, and 2001 Indian census data. We use a linear interpolation for missing years using the three census data points of 1981, 1991 and 2001. $\phi_{d}\;$ represents district level fixed effects, which account for any time invariant district characteristics. Inclusion of cohort fixed effects $\theta_{t},$ controls for any year specific shocks (political or economic). We produce heteroskedastic robust standard errors. Standard errors are clustered at the district primary sampling unit. The DLHS survey is based on a multi-stage stratified sampling design. Aggregated data for each district are based on forty Primary Sampling Units (PSUs – Villages/Urban Frame Size). These were selected with probability proportional to size (PPS) using the 1991 Census data. The number of PSUs in rural and urban areas was decided based on the proportion of urban population in a given district.

There is a possibility of measurement error in the sample due to between district migration. This may happen if a child has migrated to a different district after birth. In these cases, the value for female political representation measured at birth would not adequately map with the district environment in which they lived when surveyed between 2002–04. In the absence of information related to migration in the DLHS-2 sample, it is difficult to account for any movement of children and mothers across districts. However, evidence point at the fact that female migration in India is rather low. The primary reason of migration for women in India is marriage. Migration due to economic reasons of job search and market opportunities is predominantly for males [27, 49]. Findings based on Indian census data suggests that only 13% of the total female population migrated across districts during the 1991–2001 period [49]. Even if females migrate, due to marital reasons, the average travel time is only around 3 hours from their home villages and thus most likely within the same district [27, 49]. Given this we think that this potential source of measurement error is likely to be small.

## Results

### First stage regression

Results of our first stage estimations are reported and discussed in the S1 Appendix (Supporting Information, Table: *First stage regression*). Overall, the first stage results suggest that the instrumental variable, the proportion of seats won by women politician in a close election against male candidate, is a strong predictor for the proportion of seats won by women politician. The large F-statistics of first stage regression results appears to rule out any issue related to weak instrument.

### Second stage regression

Table 3, columns 1 and 2, displays the OLS and IV estimated coefficients for the dependent variable underweight. It takes value one if child is underweight (WAZ < −2), and zero otherwise. Columns 3 and 4 show the estimated coefficients for the dependent variable severely underweight. It takes value one if child is underweight (WAZ < −3), and zero otherwise. These dummies variables are based on the widely used WAZ estimates computed by using the WHO 2006 growth references [43]. The Z-scores are constructed using anthropometry data on weight, age, and sex using the STATA zscore06 command. To assess nutritional status of children with respect to the reference population, Z–scores (standardised scores) are employed. Z score is defined as: (observed weight of the child – median weight of the reference population)/ (standard deviation of weights in the reference population) and then standardised. The main coefficient of interest in Table 3 is the proportion of seats won by female politician in a given district when a child was born in year *t*. We have also replicated our results by considering a three-year average of the proportion of seats won by female politician. If child is born in year *t*, we consider the three-year average [(t-2) + (t-1) + (t)]/3 of the share of seats won by female politicians. We observe no changes in the key coefficient of interest. These results are presented below in Section ‘Moving average of political variables’. OLS estimates in panel A suggests that women political representation is not significantly correlated with the probability that a child is underweight, or severely underweight. Panel B to E in Table 3 display 2SLS estimates. They are reported for various specifications: panel B omits any polynomials for margin of victory or defect (forcing variable); panel C controls linearly for margin of victory or defect and we use higher order (2^nd^ and 3^rd^) polynomials of these margins in Panel D, and E respectively. We are aware that these high order polynomials are known to lead to noisy estimates in regression-discontinuity designs [50].

**Table 3:** Main results (second stage estimations) for children being underweight or severely underweight (based on WHO references).

	(1)	(2)	(3)	(4)
*Estimated coeff for prop. of seats won by female politicians*	Underweight	Severely underweight		
	0-60 months	0-24 months	0-60 months	0-24 months
**Panel A: OLS regressions**				
No individual & district controls	−0.015	−0.033	−0.006	−0.008
	(0.037)	(0.078)	(0.030)	(0.063)
*R* ^2^	0.072	0.080	0.053	0.070
With Individual controls	−0.022	−0.038	−0.011	−0.012
	(0.036)	(0.076)	(0.030)	(0.062)
*R* ^2^	0.100	0.118	0.070	0.092
With individual and district controls	−0.025	−0.054	−0.010	−0.021
	(0.036)	(0.076)	(0.030)	(0.062)
*R* ^2^	0.101	0.119	0.070	0.093
Cohort FE	yes	yes	yes	yes
District FE	yes	yes	yes	yes
**Panel B: 2SLS regressions with no electoral margins**				
No individual & district controls	−0.296^***^	−0.469^**^	−0.217^**^	−0.292
	(0.112)	(0.233)	(0.091)	(0.195)
*R* ^2^	0.072	0.080	0.053	0.070
With Individual controls	−0.306^***^	−0.424^*^	−0.221^**^	−0.271
	(0.111)	(0.230)	(0.091)	(0.195)
*R* ^2^	0.100	0.118	0.069	0.092
With individual and district controls	−0.328^***^	−0.374^*^	−0.249^***^	−0.237
	(0.109)	(0.224)	(0.089)	(0.191)
*R* ^2^	0.101	0.119	0.070	0.093
Controls for Electoral margins	No	No	No	No
Cohort FE	yes	yes	yes	yes
District FE	yes	yes	yes	yes
**Panel C: 2SLS regressions with Linear margins**				
No individual & district controls	−0.380^***^	−0.443^*^	−0.243^**^	−0.273
	(0.131)	(0.248)	(0.106)	(0.206)
*R* ^2^	0.072	0.081	0.053	0.071
With Individual controls	−0.393^***^	−0.460^*^	−0.247^**^	−0.290
	(0.129)	(0.243)	(0.106)	(0.205)
*R* ^2^	0.100	0.119	0.070	0.093
With individual and district controls	−0.388^***^	−0.424^*^	−0.261^**^	−0.266
	(0.128)	(0.243)	(0.105)	(0.206)
*R* ^2^	0.102	0.119	0.070	0.093
Controls for Electoral margins	1st Order	1st Order	1st Order	1st Order
Cohort FE	yes	yes	yes	yes
District FE	yes	yes	yes	yes
**Panel D: 2SLS regressions with 2nd Order polynomials**				
No individual & district controls	−0.379^***^	−0.468^*^	−0.252^**^	−0.307
	(0.128)	(0.247)	(0.104)	(0.206)
*R* ^2^	0.073	0.081	0.054	0.071
With Individual controls	−0.391^***^	−0.481^**^	−0.256^**^	−0.322
	(0.126)	(0.242)	(0.103)	(0.205)
*R* ^2^	0.101	0.119	0.070	0.093
With individual and district controls	−0.383^***^	−0.439^*^	−0.269^***^	−0.298
	(0.125)	(0.241)	(0.102)	(0.206)
*R* ^2^	0.102	0.120	0.071	0.093
Controls for Electoral margins	2nd Order	2nd Order	2nd Order	2nd Order
Cohort FE	yes	yes	yes	yes
District FE	yes	yes	yes	yes
**Panel E: 2SLS regressions with 3rd Order polynomials**				
No individual & district controls	−0.313^**^	−0.491^*^	−0.189^*^	−0.317
	(0.129)	(0.273)	(0.104)	(0.228)
*R* ^2^	0.073	0.082	0.054	0.071
With Individual controls	−0.326^**^	−0.515^*^	−0.193^*^	−0.339
	(0.127)	(0.268)	(0.104)	(0.227)
*R* ^2^	0.101	0.119	0.070	0.093
With individual and district controls	−0.314^**^	−0.470^*^	−0.205^**^	−0.314
	(0.126)	(0.266)	(0.103)	(0.227)
*R* ^2^	0.102	0.120	0.071	0.094
Controls for Electoral margins	3rd Order	3rd Order	3rd Order	3rd Order
Cohort FE	yes	yes	yes	yes
District FE	yes	yes	yes	yes
Observations	122926	46349	122926	46349

Note: Robust standard errors clustered at districts primary sampling unit reported in parentheses. All regression equations include cohorts and district fixed effects. Individual controls include child gender, child age in months, mother age at childbirth, mothers’ education level, caste (gen, SC, ST), religion (Hindu, Muslim), live in rural areas, and Socio-economics status index. District level controls include female population, urban population, SC/ST population, and literacy rate. Also, all 2SLS regressions are controlled for the proportion of constituencies in a district that had total close elections between women and men. ^*^ *p* < 0.1, ^**^ *p* < 0.05, ^***^ *p* < 0.01

In column 1, for the sample of 0–60 months old children, the coefficients of women representation are consistently negative and statistically significant. As shown in panels B to E, results are robust to conditioning on different set of controls and on various functional forms of margin of victory or defeat polynomials. The magnitude of the coefficient in the fullest specification, when controlling for 3^rd^ order polynomial in panel E, suggests that while holding the other factors constant, a rise in women representation by 10 percentage points would significantly reduce the probability of children under five being underweight by approximately 3.1 percentage points.

For the cohort of 0–24 months underweight children in Column (2): the magnitude of the coefficient of share of seats won by female politician is negative and statistically significant. It implies that by increasing the share of female political representation by 10 percentage points, the probability that children aged 0–24 months will be underweight (malnourished) in a district decline by approximately 4.7 percentage points (panel E of columns 2) and is statistically significant. However, the level of significance varies across different set of specifications, but key coefficients remain statistically significant.

We investigate the effect of women political representation for severely underweight children aged 0–60 months in column 3 and aged 0–24 months in column 4. These children face significant risks of deaths [51]. The IV coefficient for severely underweight children is negative, and consistently statistically significant across panels for the 0–60 months subsample only. Column 4 for the subsample of 0–24 months shows no significant impact. The coefficients in panel E indicates that by increasing the share of female political representation in a district by 10 percentage points, the probability that children aged 0–60 months will be severely underweight declines significantly by about 2 percentage points.

The OLS coefficients are smaller than the 2SLS ones. This downward bias may come from the fact that the omitted variable (voters’ preferences) is positively correlated with female representation and negatively correlated with the likelihood of malnutrition (or vice and versa). Constituencies with high malnutrition rates will tend to elect female politicians if they are viewed as the right candidates for improving nutritional outcomes. A similar downward bias is observed in [29]. It may come from the fact that female politicians who win in close election tend to act differently, in a more effective manner, in terms of policy choices and delivery in comparison to the average elected female politicians.

A plausible explanation for this negative effect may be that female politicians tend to focus on and develop more policies to promote early childhood health and development. [26] shows that larger female political representation reduces neonatal mortality as they tend to invest more in village level health infrastructure. Literature in the field of early childhood development points at the importance of offering health and nutrition interventions at an early age [52]. Malnutrition in the early stages can lower children’s resistance to diseases, infections, and increases the risk of morbidity and mortality among children. We present an analysis of the possible channels through which this effect takes place in Section ‘Discussion on channels’.

An alternative way of getting WAZ estimates consists of using the National Centre of Health Statistics (NCHS) growth references provided by DLHS survey estimates. Z score is then defined as: (observed weight of the child – median weight of the reference population given by the DLHS survey)/ (standard deviation of weights in the reference population given by the DLHS survey). We construct equivalent dependent dummy variables, as used in Table 3, but by using these alternative WAZ estimates: one if child is underweight (WAZ < −2), and zero otherwise; one if child is severely underweight (WAZ < −3), and zero otherwise. Estimations results are broadly similar to the ones displayed above. These are shown and discussed in the S1 Appendix in Table S3.1.

Table S3.2 (in S1 Appendix) presents the 2SLS estimates based on the restricted sample of districts-year observations which display at least one election race between both a female and a male candidate. The dependent dummy variables are computed by using the WHO 2006 growth reference. It is possible that these districts behave differently and that results for them differ to baseline ones. For children 0–60 months, shown in column 1, the coefficients are consistently negative and significant and robust to various sets of controls which is in line with the baseline results. Looking at younger cohort (column 2), no significant negative effects are detected. Likewise, we detect no significant effect of women’s representation when we use the severely underweight dummy as dependent variable in column 3.

Table 4 presents the 2SLS estimates based on the continuous dependent variables weight-for-age z-scores (WAZ). The WAZ estimates in columns 1 and 2 are based on the WHO 2006 growth references. Results in the columns 3 and 4 are based on National Centre of Health Statistics (NCHS) growth reference provided by DLHS survey estimates. Weight-for-age z-scores in column 1 and 2 in Table 4 are based on our own calculation using the WHO guideline 2006 reference. The Z-scores are constructed using anthropometry data on weight, age, and sex using the STATA zscore06 command. The weight-for-age z-score in column in 3 and 4 in Table 4 are based on the National Centre for Health Statistics (NCHS) growth reference provided by DLHS estimates. The use of NCHS base makes the results internationally comparable.

**Table 4:** Regression results using the continuous Weight-for-age (WAZ) z-score.

	(1)	(2)	(3)	(4)
	WAZ (WHO standards 2006)		WAZ (as per NCHD reference)	
*Estimated coeff for prop. of seats won by female politicians*	0-60 months	0-24 months	0-60 months	0-24 months
**Panel A: 2SLS regressions with no electoral margins (no polynomials)**				
No individual & district controls	0.410	0.969	0.950^***^	0.592
	(0.355)	(0.862)	(0.342)	(0.812)
*R* ^2^	0.113	0.116	0.144	0.188
With Individual controls	0.468	0.854	1.003^***^	0.519
	(0.349)	(0.842)	(0.335)	(0.772)
*R* ^2^	0.152	0.170	0.182	0.284
With individual and district controls	0.649^*^	0.723	1.133^***^	0.301
	(0.341)	(0.826)	(0.328)	(0.758)
*R* ^2^	0.153	0.171	0.183	0.285
Controls for Electoral margins	No	No	No	No
Cohort FE	yes	yes	yes	yes
District FE	yes	yes	yes	yes
**Panel B: 2SLS regressions with Linear margins**				
No individual & district controls	0.556	0.797	1.302^***^	0.413
	(0.415)	(0.960)	(0.402)	(0.914)
*R* ^2^	0.114	0.117	0.146	0.189
With Individual controls	0.632	0.967	1.377^***^	0.757
	(0.407)	(0.932)	(0.393)	(0.863)
*R* ^2^	0.153	0.171	0.184	0.285
With individual and district controls	0.715^*^	0.847	1.404^***^	0.545
	(0.402)	(0.933)	(0.388)	(0.865)
*R* ^2^	0.154	0.172	0.185	0.286
Controls for Electoral margins	1st Order	1st Order	1st Order	1st Order
Cohort FE	yes	yes	yes	yes
District FE	yes	yes	yes	yes
**Panel C: 2SLS regressions with 2nd Order polynomials**				
No individual & district controls	0.568	0.902	1.268^***^	0.491
	(0.404)	(0.962)	(0.390)	(0.922)
*R* ^2^	0.114	0.117	0.147	0.190
With Individual controls	0.639	1.094	1.338^***^	0.908
	(0.396)	(0.934)	(0.382)	(0.868)
*R* ^2^	0.153	0.171	0.185	0.285
With individual and district controls	0.718^*^	0.960	1.359^***^	0.673
	(0.392)	(0.932)	(0.378)	(0.868)
*R* ^2^	0.155	0.172	0.186	0.286
Controls for Electoral margins	2nd Order	2nd Order	2nd Order	2nd Order
Cohort FE	yes	yes	yes	yes
District FE	yes	yes	yes	yes
**Panel D: 2SLS regressions with 3rd Order polynomials**				
No individual & district controls	0.374	0.925	1.050^***^	0.45
	(0.408)	(1.060)	(0.393)	(1.015)
*R* ^2^	0.115	0.117	0.148	0.190
With Individual controls	0.449	1.171	1.123^***^	0.944
	(0.400)	(1.033)	(0.385)	(0.961)
*R* ^2^	0.154	0.171	0.185	0.286
With individual and district controls	0.532	1.036	1.159^***^	0.697
	(0.396)	(1.027)	(0.382)	(0.957)
*R* ^2^	0.155	0.172	0.187	0.286
Controls for Electoral margins	3rd Order	3rd Order	3rd Order	3rd Order
Cohort FE	yes	yes	yes	yes
District FE	yes	yes	yes	yes
**Observations**	122926	46349	122926	46349

Note: see note at the bottom of Table 3

The estimation methods used for the WAZ outcome is similar to the strategy employed in the previous sections when used binary variables. The key coefficient of interest is once again the proportion of seats in a district held by female politicians. For children aged under five (0–60 months), Table 4 column 1 and column 3, the women representation effect is positive on weight-for-age z scores. The coefficient of WAZ based on the WHO 2006 growth reference is positive, and statistically insignificant. The coefficient of WAZ based on the DLHS estimates (national references – NCHS) is positive and statistically significant. The richest specification, column 3 in panel D, suggests that while holding the other factors constant, increasing female political representation by 10 percentage points would improve the weight-for-age z-score by around 0.1 point. For the younger cohort of 0–24 months children, columns 2 and 4, the magnitude of this effect is smaller but not significant for all specifications. These results are indicative of greater impact on weight-for-age z-score for older children (0–60 months). A plausible explanation may be that policies implemented to promote early childhood health and development take time to bring noticeable improvements. Rapid changes in weight-for-age z score of children following election of women politician is unlikely. Table 3 shows relatively low levels of explanatory power for our various models ranging from around 5–10 percent. Table 4 shows that this power is increased to levels varying from 11 to 18 percent when using continuous dependent variables. Adding district controls to our specifications bring barely any change in the explanatory power while individual controls do.

## Discussion on channels

Female politicians’ impact on children undernutrition could come from their influence on policies related to underlying causes of malnutrition. Previous work has identified the immediate and underlying causes of malnutrition: child heath, dietary diversity, household food security, care for children and mothers, and health environment. [30] provides evidence that, among others, safe hygiene and sanitation practices, availability of health care facilities and healthy environmental factors improve child nutrition outcomes. Thus, we investigate the influence of female political representation on a range of related indicators: 1) antenatal and delivery care; 2) postnatal care, child feeding practises, and caregiver awareness and 3) household access to basic amenities. Our estimation strategy is similar where close election is used as an instrument for the proportion of seats in a district won by female politician.

### Health services and caregiver behaviour

For this subsection we use the DLHS-2 mother level data which is based on a survey section that focuses exclusively on women’s last birth if it occurred between 1999 and 2004.

### Antenatal and delivery care

Results in column 1 of Table 5 indicate that an increase of ten percentage points in female political representation increases the probability that a woman received three or more antenatal check-ups during pregnancy by approximately by 2.8 percentage points. There is a high prevalence of anaemia among pregnant women in India [53]. [54] identifies that iron deficiency during pregnancy is associated with higher risks of low birth weight and adverse undernutrition outcomes. Our results, shown in column 3, suggest that female politician representation raises the probability of taking iron supplementation by pregnant women. Female representation increases the probability of institutional delivery (but mildly as only significant in Panel C), or delivery attended by a health professional (in all panels). Table 5 also shows that female political representation has no significant impact on six other antenatal variables: receiving one tetanus injection, IFA tablets (or iron tablets) and receiving various prenatal and postnatal types of advice.

**Table 5:** 2SLS regression results for prenatal and delivery care.

*Estimated coeff for prop. of seats won by female politicians*	(1)	(2)	(3)	(4)	(5)	(6)	(7)	(8)	(9)
	Received 3 or more antenatal check ups	Woman had at least one TT injection during pregnancy	Received Iron and folic tables during pregnancy	Received 100 or more IFA tablets during pregnancy	Received advice during pregnancy on delivery care	Received advice during pregnancy on new-born care	Child delivery at public inst.	Home delivery attended by a health prof.	ANM visited within 2 weeks of delivery
Mean of dependent variable	0.65	0.80	0.63	0.19	0.26	0.20	0.19	0.48	0.13
SD	0.47	0.39	0.48	0.39	0.44	0.40	0.40	0.50	0.33
**Panel A: 2SLS regressions with no electoral margins**									
No individual & district controls	0.285^*^	0.069	0.311^**^	0.125	0.123	−0.045	0.180	0.283^**^	0.139
	(0.170)	(0.117)	(0.136)	(0.103)	(0.109)	(0.098)	(0.111)	(0.136)	(0.100)
With individual and district controls	0.217	0.024	0.271^**^	0.099	0.069	−0.084	0.146	0.158	0.124
	(0.162)	(0.110)	(0.129)	(0.100)	(0.104)	(0.094)	(0.108)	(0.120)	(0.098)
**Panel B: 2SLS regressions with Linear margins**									
No individual & district controls	0.303^*^	0.058	0.313^**^	0.118	0.117	−0.047	0.173	0.289^**^	0.136
	(0.170)	(0.122)	(0.140)	(0.108)	(0.114)	(0.102)	(0.115)	(0.143)	(0.104)
With individual and district controls	0.231	0.013	0.274^**^	0.09	0.057	−0.092	0.138	0.153	0.130
	(0.165)	(0.117)	(0.136)	(0.106)	−0.111	(0.100)	(0.113)	(0.129)	(0.104)
**Panel C: 2SLS regressions with 3rd Order polynomials**									
No individual & district controls	0.286^*^	0.084	0.328^**^	0.073	0.072	−0.104	0.193^*^	0.272^*^	0.145
	(0.167)	(0.124)	(0.141)	(0.107)	(0.113)	(0.100)	(0.113)	(0.142)	(0.102)
With individual and district controls	0.220	0.038	0.289^**^	0.047	0.010	−0.147	0.159	0.142	0.132
	(0.163)	(0.119)	(0.137)	(0.105)	(0.110)	(0.099)	(0.112)	(0.128)	(0.102)
Observations	80815	108714	108709	108709	108689	108690	108315	108746	108746

Note: see note at the bottom of Table 3

### Postnatal care, child feeding practises, and caregiver awareness

The impact of female political representation on the probability that a woman has received healthcare treatment after childbirth is positive and statistically significant, see Table 6 column 1 and 2. [55] suggests that breastfeeding can improve cognitive development and bonding of children with mother. Breastfeeding can also protect new-born against major causes of diseases and infections [56]. The coefficient for breast-feeding within 24 hours of childbirth is not significant (column 3). The first two years of a child life is crucial: child caregiver’s knowledge and nutrition during this window reduces morbidity and mortality among children [52,57]. Our results suggest that the coefficient for the dependent variables for awareness of the danger signs of diarrhoea (column 4) is mildly significant. When we look at ORS awareness (ORS hydration salt), awareness of signs of pneumonia and diarrhoea management among mothers, female political representation appears to have no statistically significant impact (column 5, 6 and 7). However, we observe a positive and statistically significant impact on the likelihood of children receiving treatment for pneumonia (column 8).

**Table 6:** 2SLS regression results for postnatal care, child feeding practice, and caregiving awareness.

*Estimated coeff for prop. of seats won by female politicians*	(1)	(2)	(3)	(4)	(5)	(6)	(7)	(8)
	Post delivery: woman with complication received treatment	Post delivery: received treatment for health problem	Immediately breastfeed after birth	Aware of danger signs of diarrhoea	Aware of ORS (hydration salt)	Received treatment for children with diarrhoea	Aware of danger signs of pneumonia	Received treatment for children with Pneumonia
Mean	0.05	0.15	0.28	0.44	0.34	0.15	0.44	0.03
SD	0.21	0.36	0.45	0.50	0.47	0.37	0.50	0.16
**Panel A: 2SLS regressions with no electoral margins (no polynomials)**								
No individual & district controls	0.153^**^	0.493^***^	0.069	0.207	0.12	0.025	0.145	0.077^**^
	(0.064)	(0.171)	(0.111)	(0.141)	−0.158	−0.247	−0.138	−0.038
With individual and district controls	0.143^**^	0.442^***^	0.059	0.106	0.026	0.015	0.115	0.074^**^
	(0.063)	(0.167)	(0.109)	(0.129)	−0.148	−0.243	−0.133	−0.038
**Panel B: 2SLS regressions with Linear margins**								
No individual & district controls	0.172^**^	0.580^***^	0.026	0.295^**^	0.153	−0.036	0.13	0.079^**^
	(0.067)	(0.189)	−0.117	−0.147	−0.161	−0.276	−0.143	−0.04
With individual and district controls	0.161^**^	0.525^***^	0.011	0.182	0.054	−0.037	0.094	0.077^*^
	(0.067)	−0.188	−0.116	−0.137	−0.152	−0.275	−0.139	−0.04
**Panel C: 2SLS regressions with 3rd Order polynomials**								
No individual & district controls	0.165^**^	0.546^***^	0.015	0.303^**^	0.15	−0.073	0.114	0.070^*^
	(0.067)	(0.195)	−0.117	−0.147	−0.157	−0.286	−0.142	−0.04
With individual and district controls	0.158^**^	0.488^**^	0.004	0.185	0.052	−0.076	0.082	0.067^*^
	(0.067)	−0.194	−0.116	−0.137	−0.149	−0.286	−0.139	−0.04
Observations	108746	34096	108772	108772	88935	15352	108739	108670

Note: see note at the bottom of Table 3.

Overall, an increase in female political representation appears to have a positive impact on a subset of antenatal and postnatal health seeking behaviour. These findings are consistent with [26] which show, based on different data, that female politician representation in India improves public provision of antenatal and childhood health services.

### Household access to basic amenities

Access to clean water, sanitation, and hygiene is also considered an underlying factor to improve undernutrition among children [30]. Female politicians tend to favour policies that directly facilitate access for households to safe fuel, water, and a safer environment. [21] shows that in local government, women leaders (*village Pradhan*) invest more in village level public goods that are linked to the women’s concerns such as drinking water. We carry out a similar investigation to see whether female political representation at the district level can affect households’ access to basic goods such as access to clean energy, clean water, and sanitation. For this we use household information from the DLHS-2 survey. A sample of 394,505 households were surveyed between 2002 and 2004 on this theme. We look at six different household amenities as dependent variables. The specification and estimation strategy are similar to the baseline analysis for our nutrition outcome.

Table 7 presents the effects of female political representation on basic amenities at the household level. Apart from access to LPG clean cooking fuel (column 6), results indicate that an increase in female political representation has a significant and positive effect on household access to relevant amenities. Column 1 suggests that increasing female politician by 10 percentage points significantly increases the likelihood of households having access to clean water and tap water by 6.9 and 9.6 percentage points, respectively. Likewise, we observe a strong and positive effect on household access to electricity (column 3), on access to toilet facilities at home (column 4) and shared public toilet facilities (column 5).

**Table 7:** 2SLS regression results for effects on household basic amenities.

*Estimated coeff for prop. of seats won by female politicians*	(1)	(2)	(3)	(4)	(5)	(6)
	Access to clean water	Access to tap water	Access to electricity	Access to improved toilet facilities	Access to toilet facility (including shared/public toilet)	Access to cooking fuel: LPG
Mean	0.81	0.43	0.67	0.43	0.40	0.26
SD	0.39	0.50	0.47	0.50	0.49	0.44
**Panel A: 2SLS regressions with no electoral margins (no polynomials)**						
No individual & district controls	0.643^***^	0.966^***^	0.438^***^	0.422^***^	0.405^***^	0.102
	(0.086)	(0.114)	(0.096)	(0.121)	(0.116)	(0.107)
With individual and district controls	0.561^***^	0.860^***^	0.239^***^	0.224^***^	0.206^***^	0.047
	(0.082)	(0.102)	(0.06)	(0.055)	(0.053)	(0.04)
**Panel B: 2SLS regressions with Linear margin**						
No individual & district controls	0.792^***^	1.183^***^	0.450^***^	0.461^***^	0.444^***^	−0.065
	(0.123)	(0.162)	(0.135)	(0.171)	(0.164)	(0.151)
With individual and district controls	0.729^***^	1.133^***^	0.273^***^	0.327^***^	0.304^***^	0.008
	(0.116)	(0.142)	(0.085)	(0.078)	(0.075)	(0.056)
**Panel B: 2SLS regressions with 3rd Order polynomials**						
No individual & district controls	0.695^***^	0.966^***^	0.408^***^	0.400^**^	0.407^***^	−0.086
	(0.114)	(0.114)	(0.124)	(0.157)	(0.15)	(0.139)
With individual and district controls	0.695^***^	0.966^***^	0.408^***^	0.400^**^	0.407^***^	−0.086
	(0.114)	(0.114)	(0.124)	(0.157)	(0.15)	(0.139)
Observations	394505	394505	394505	394505	394505	394505

Note: see note at the bottom of Table 3

[58, 59] show that approximately fifty percent of all forms of malnutrition are associated with continuous occurrence of diarrheal or intestinal infection because of inadequate access to safe drinking water, hygiene, and sanitation. For young children, diarrhoea can cause permanent damage to intestinal development, lowering the child’s ability to absorb nutrients. Improvement in sanitation, especially in eliminating open defecation, help to reduce stunting in rural areas by 4–37 percent [60]. This literature acknowledges the importance of access to improved water, sanitation, and hygiene facilities as an important mechanism to reduce malnutrition among children. Studies document a strong and positive relationship between access to electricity and food security at the household level and that energy poverty is associated with poor health outcomes [61, 62]. [63] finds that households’ access to electricity significantly improves children’s health outcomes in India.

Our evidence indicates that a larger share of female politicians at district level can improve environmental factors at the community and household level such as access to safe drinking water, tap water, toilet facilities and electricity. These in turn can improve nutrition outcomes for children. Access to food is not enough for communities that are prone to water related infections which are spread because of poor sanitation facilities. Proper sanitation facilities and safe drinking water can reduce under-nutrition by preventing diseases and infections.

### Robustness checks

#### Alternative thresholds for close elections.

For identification strategy, the outcome of a close election can be considered quasi-random [45]. This holds for narrow vote margins between winner and the runner-up. There is an efficiency trade-off in choosing the cut-off point or width of this vote margin. Some studies related to Indian elections data argue that elections with up to a 5% victory margin can be interpreted as close election [64–66]. For the sake of completeness, we conduct robustness-checks using alternative definitions where the margin of victory between winner and runner-up of opposite gender is set at different cut-off points: 2%, 2.5%, 3%, 4%, 4.5%. 5%, 5.5% and 6%.

Results in Table S4 panel A are based on 2SLS estimates with no electoral margins polynomial for the dependent dummies of underweight and severely underweight children using various alternative thresholds for close election. Results are reported without controls, with individual controls, and with individual and district level controls for children under five (in column 1–3 and 7–9), and children under two (in column 4–6 and 10–12). Overall results show some variations in the coefficient of proportion of seat won by the female politician using different victory margin cut-offs. Results appear robust to conditioning on different set of specifications by using higher order polynomials of margin of victory or defeat presented in Table S4 panel B and C in the S1 Appendix. There is no important change in the coefficient of female political representation across all age cohorts in comparison to our main results presented in Table 3. The dependent dummy variables are based on the 2006 WHO child growth standards reference.

Similarly, we use the continuous variable (WAZ) for robustness-checks using these alternative thresholds. For the cohort of 0–60 months children, in column 1–3 of Table S5 and based on the WHO growth reference 2006, we observe that at the narrowest margin of 2% the coefficient is positive but not statistically significant. It is at the 4% threshold or above, that the effect of female politician consistently becomes statistically significant. These results offer a different conclusion than the one from Table 4: overall there seems to be a trend showing a statistically significant impact of female representation on weight-for-age z score. Results for WAZ based on the national growth reference (NCHD) (in column 7–9) show that for children under five there is a strong, positive, and statistically significant impact of female representation on weight-for-age z score across all victory margin thresholds. These results are consistent with the results presented in Table 4. For the younger cohort aged 0–24 months, the results are presented in Table S5 in columns 4–6 and 10–12. They suggest that at the narrowest vote margin of 2% and 2.5% the coefficient of female representation is positive and either mildly significant at 10% or not. Once again, at 4% and above threshold of vote margin, the results are consistently positive and statistically significant. Results appear robust to conditioning on different set of specifications by using higher order polynomials of margin of victory or defeat presented in Table S5 panel B and C in the S1 Appendix.

### Moving average of political variables

The main analysis presented above is done with merging DLHS-2 data and election data at child’s birth year. This means that for a 5-year-old child surveyed in 2002 we merge the information on that child from the DLHS-2 with the corresponding data on women’s political representation at the district level for the year 1997. Arguable policy decisions made by the politicians in power may take time to impact their electorates. In our specifications, their impact would operate with lags. Using a moving average of a few years for the political variables provides a worthwhile robustness check while keeping a similar identification strategy. We consider a moving average of three years of the proportion of seats won by female politician prior and including when a child was born. For our 5-year-old child surveyed in 2002 we now merge the corresponding political data on for the years 1995, 1996 and 1997. Women’s political representation at the district level is thus the average over these three years. Similarly, we use three-years averages for district level control variables.

The estimates based on three year moving average of all political variables and an election threshold of 3.5% are reported in Table S6 in the S1 Appendix. For the dependent variables underweight and severely underweight and the sample of children under 60 months: results are in line with the main ones we present in Table 3. However, for younger cohort (under 24 months): relevant coefficients (in columns 4) remain insignificant when using the dependent variable severely underweight as in Table 3. When using underweight (column 2), the coefficients for female representation lose some of their significance. Overall, estimates based on the moving average of three years of the proportion of female politician in a district are consistent with the baseline results.

### Placebos and lags

Placebo checks are done to mitigate the problem that we may be erroneously capturing omitted trends. For this, we estimate the same model as in Table 3 for the dependent dummy variable whether a child is underweight or not. For each cohort of children born in year *t* we use future female political representation by considering leads for up to 5 years after the childbirth (*t + 1*, *t + 2*, *t + 3*, *t + 4*, and *t + 5*). To allow sufficient sample sizes we carry this placebo analysis for children aged 0–24 months. For this sample it is likely that a child who is surveyed while aged 12–24 months may still be impacted by female political representation during the first year of her life (*t + 1*). We would expect this impact to be insignificant for additional leads: *t + 2* up to *t + 5*. For the sample of children aged 0–60 months further leads starting from *t + 5*, *t + 6, t + 7* and so on would be needed. Our political data extend up to 2007 and as such do not allow us to carry this analysis. Our data on political representation extend to 2007 and allow us this number of leads (children in our sample are born between 2002 and 2004). Results from Table S7, in the Supporting Information S1 Appendix, show that the coefficient of female representation for *t + 1* is mildly positive and significant but those for further leads are all essentially zero.

It is important to recall that we do not know whether the previous location of households (for any lag) was in the same district or in a different one. This adds noise to the estimates. This issue, due to lack of information on migration, is also acknowledged in other relevant analysis [26].

### Mothers’ fixed effects, heterogeneity, and outliers

We have a subsample of 27252 mothers who have more than one child included in our sample. This represents 57031 children aged 0–60 months and 2477 aged 0–24 months. We extend our analysis by adding mothers’ fixed effects. Mother level fixed effects will account for any time invariant preferences of female which may differ across mothers. In the results presented so far, we already control for the mother’s age at child’s birth in the regression which will vary by birth cohort of the child. The estimates based on this subsample of children are reported in Table S8 in S1 Appendix. For children 0–60 months of age, results differ little compared to the baseline ones. However, the impact of female political representation observed in our baseline results seem to vanish for children 0–24 months.

As an additional robustness check, we restrict our sample by omitting what could be considered as outliers in women’s representation above the seventy-fifth percentile. This amounts to removing the 4.17 percent of observations with women representation proportion greater than 25 percent. Results based on this subsample are shown in Table S9 (S1 Appendix). They are very much in line with the main ones discussed above in Table 3. We also explore heterogeneous impacts based on gender, rurality, caste, and marital status in Table S10 in S1 Appendix. These results are similar to the baseline results for children aged under five (0–60 months). However, for the sample of children aged 0–24 months, we find little impact of female political representation.

A key identification assumption is that the outcome of close elections between male and female candidates is quasi-random. In RD design, the necessary condition for identification is that of continuity of conditional expectation of counterfactual in the forcing variable. The continuity assumption may not hold if there is some manipulation of the forcing variable. Specifically, a potential threat to the identification assumption could come from vote manipulation. A visual inspection of the density of vote margins rejects this: see Supporting Information Section S1.5.

## Conclusion

Evidence from previous studies demonstrates positive impact of female politicians on education, neonatal mortality, and female labour market opportunities. Our results contribute to the literature by showing a significant and positive impact of female political representation on child malnutrition status. Female political representation at district level appears to reduce malnutrition rate when looking at samples of children under five or under two. Supporting the existing literature on the benefits of political agency of women, our analysis indicates that increasing female representation in the elected governments could be an effective way to reduce malnutrition among children in developing countries. Where the role of gender preferences of elected leaders can play an important part, such as appears to be the case for malnutrition, they could bring benefits along that metric. It improves households’ access to basic amenities and some aspects of prenatal, delivery and postnatal care among mothers.

Our findings suggest the importance of empowering female leaders and their potential in transforming women’s and children’s lives. Although Indian legislature has recently passed Women’s Reservation Bill 2023 to reserve one-third elected members in the lower house and state assemblies for females, there are several challenges and expected delays in implementation. Given the evidence from our findings of the benefits of increased female representation, we contribute to the argument of timely implementation of the bill.

## Supporting information

S1 Appendix This appendix provides further details on the selection of the data used in the study, various robustness checks, and numerous figures. (DOCX)
