# Supplementary material for: Malnutrition and female political representation in India
Source: PLoS One. 2026 Mar 18;21(3):e0342588. doi: 10.1371/journal.pone.0342588 (PMC12998867; doi:10.1371/journal.pone.0342588)
Supplement: S1 Appendix — (DOCX) [file pone.0342588.s001.docx]

**SUPPORTING INFORMATION**

**S1 Appendix**

**CONTENT:**

**-Section S1: pages 1-10**

**-Tables ‘First stage regression’ and S3.1 to S9: pages 11-33**

**Section S1:**

**Selection of Dataset**

Our research focuses on children aged 0 to 5 years since early investments during this period are fundamental to human capital development. In India, two major health surveys provide anthropometric data: the District Level Health Survey (DLHS) and the National Family Health Survey (NFHS). We selected DLHS-2 for this analysis based on the following considerations:

**1. District-Level Representativeness**

DLHS is a district-representative survey providing comprehensive anthropometric outcomes in India (discussed in Section ‘Nutrition data’). This district-level granularity is essential for our analysis, as it allows us to examine variation across 563 districts, of which 361 were successfully matched with electoral data (discussed in Section ‘Sample selection […]’).

**2. DLHS-2 over DLHS-3; DHLS-4**

We used DLHS-2 (2002-04) rather than DLHS-3 (2007-08) because anthropometric data was not collected in the third round (this is mentioned in Section ‘Sample selection […]’).

DLHS-4 (2012-13): Although preliminary reports were released in 2016-17, the whole dataset became publicly available only in 2020, after we had completed our lengthy electoral data collection for the 1993-2004 period.

**3. Considering other datasets:**

**IHDS** (Indian Human Development Survey), a panel survey conducted by the University of Maryland in 2004-05 and 2011-12, was considered but ultimately excluded for these reasons:

1. Sample Size Constraints**:** After constructing height/weight z-scores for children under five years —our focal age group— many districts had insufficient observations with typically fewer than 12 per district, making robust statistical inference impossible.
2. Pooling IHDS-I and IHDS-II proved difficult due to the Delimitation Act of 2002. It led to the creation of new constituencies and dissolution of others and to changes in constituency reservation status for socially disadvantaged candidates.

**NFHS** rounds 1, 2, and 3 are state-representative surveys that do not permit district identification. For our analysis a district representative sample is required.

**NFHS-4 (2015-16):** Released nearly a decade after NFHS-3, this round introduced district-level representativeness: a significant methodological improvement. However, the full dataset became available much after we started the empirical work presented in the manuscript and the lengthy electoral data collection we had completed at that stage.

More critically, using **DHLS-4** and **NFHS-4** can present with a potential problem. The 2002 Delimitation Act modified several constituencies boundaries. These changes were made, in a staggered fashion across states, from 2008 onwards and as such do not impact the period covered by DLHS-2 and our empirical analysis. Most Indian states held their first elections under these new boundaries between 2009-2011. This act reserved a number of seats for Schedules Castes and Scheduled Tribes. This reservation can potentially lead to bias in the gender representation which would have complicated the empirical analysis. To avoid this issue, one would need to restrict the analysis to children aged 0-24 months for the DLHS-4, limiting the scope of the analysis. This would have impacted similarly the use of NFHS-4.

**NFHS-5 (2019-21):** The full dataset became available after we had completed this paper which took several years. Additionally, given its timing, researchers planning to use this dataset will have to deal with the confounding effects of the COVID-19 pandemic (2019-2021).

**HUNGAMA Study (2011):** This survey covers only India's 100 poorest districts, limiting generalizability and geographic scope.

**Annual Health Survey (AHS, 2013 onwards):** Available only in aggregated form, preventing individual-level analysis required for our research design.

Given the above-mentioned points and when we started and progressed with this research project, DLHS-2 emerged as the optimal dataset. It allows us to examine political and nutritional outcomes within stable institutional boundaries (districts and states), providing a clean identification strategy.

**Regression discontinuity: identification validity**

**Figure S1:**

RD plot of share of seats won by female politicians with respect to margin of victory

**
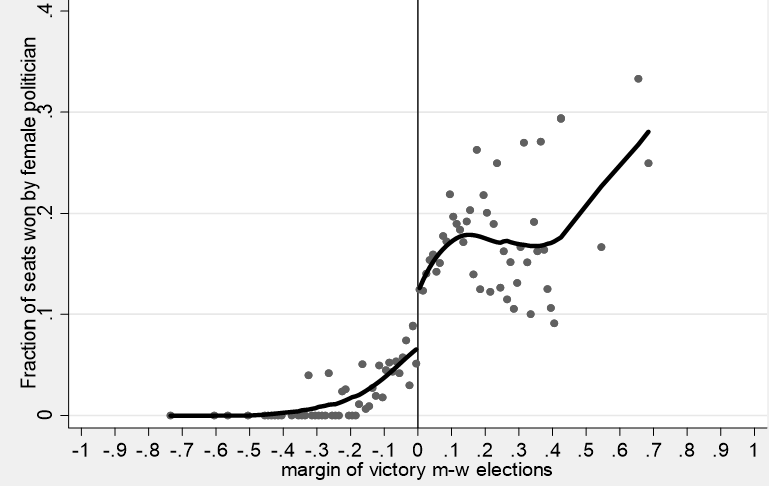
**

The sharp jump at the discontinuity threshold of zero suggests that female winning in a close election in a district increases the overall share of seats won by female politicians in a district by roughly 7 percentage points.

*S1.1 Randomness of the outcome of a close election*

We provide evidence supporting the randomness of the outcome of a close election by running an OLS where political and demographic characteristics are used to predict the probability that women win in close elections (when faced against men) in the district. If the outcome of close elections between a male and a female politician is impacted by these variables: the gender of the winners cannot be considered random. The dependent variable is the proportion of close elections won by women by district in each electoral year. This is regressed by a host of political and demographic variable at the district level shown in Table S1.1. Results from that table show that none of the coefficients is individually significant and all 7 demographic and political variables (excluding year and district fixed effect) are jointly non-significant. This suggests that the outcome of a close election can indeed be considered as good as random.

**Table S1.1:** Explaining the proportion of women winning in close elections when against men.

|  | (1) |
| --- | --- |
| Sample election: 1993 to 2004 | Prop. of women winning in close elections when against men;  per district and electoral year. |
| Total number of male and female elections in the previous election year | -0.056 (0.266) |
| No. of elections a woman has won in the district in the previous election year | -0.319 (0.480) |
| Proportion of seats reserved for SC/ST candidates | -22.858 (38.374) |
| Proportion of urban population | 11.152 (31.212) |
| Proportion of SC/ST population | 66.214 (81.880) |
| Proportion of female population | 72.824 (219.275) |
| Proportion of literacy rate  Year FE  District FE | 10.033 (15.216)  Yes  Yes |
| Observations | 161 |
| *R*^2^ | 0.949 |

Standard errors in parentheses. Robust SE clustered at the district level.

^*^ *p* < 0.1, ^**^ *p* < 0.05, ^***^ *p* < 0.01

F(7, 130) = 0.84 (p-value of 0.56) for all controls except year and district FE.

*S1.2 Comparing along observables*

We show that the districts in which female candidates won in close elections against men are similar along observables to those in which male candidates won in close elections against women. Table S1.2 shows no statistically significant differences in districts characteristics of districts (at 10% level) where more female politicians win in close elections than male politicians, compared to districts where more male politicians win in close elections than female politicians. For this, we restricted the sample to districts that had at-least one close constituency election between women and men. This subsample of districts consists of two categories: group one gathers districts where more women politicians won in close elections against men politicians; and group two gather districts where more men politicians won in close elections against women politicians. P-values for three characteristics are slightly above 10% (Urban population, SC and ST population and Female literacy rate). On the whole means in both samples are very much in line, the small SE are what drives down p-values.

**Table S1.2:**

Comparing close election districts won by women politicians against men politicians.

|  | **Close election won by women** | | **Close election won by men** | | **Difference** | | **P-value** |
| --- | --- | --- | --- | --- | --- | --- | --- |
|  | **mean** | **SE** | **mean** | **SE** | **Mean** | **SE** |  |
| Urban Population | 0.291 | 0.015 | 0.258 | 0.014 | -0.033 | 0.021 | 0.109 |
|  |  |  |  |  |  |  |  |
| SC and ST Population | 0.223 | 0.007 | 0.241 | 0.009 | 0.019 | 0.012 | 0.111 |
|  |  |  |  |  |  |  |  |
| Female Population | 0.484 | 0.001 | 0.483 | 0.001 | -0.001 | 0.002 | 0.552 |
|  |  |  |  |  |  |  |  |
| Male literacy rate | 0.375 | 0.007 | 0.370 | 0.006 | -0.005 | 0.009 | 0.589 |
|  |  |  |  |  |  |  |  |
| Female literacy rate | 0.267 | 0.008 | 0.250 | 0.007 | -0.017 | 0.011 | 0.113 |
|  |  |  |  |  |  |  |  |
| Labour participation | 0.237 | 0.010 | 0.224 | 0.009 | -0.013 | 0.014 | 0.340 |
| District-year obs | 160 |  | 205 |  |  |  |  |
| Total no. of districts with close election | 106 |  |  |  |  |  |  |

*S1.3 External validity*

We provide evidence that districts that had close elections (at 3.5%) between a man and a woman are not systematically different from other districts in India. Table S1.3 exhibits mean differences of district demographic characteristics that had close elections between female and male politicians, and all other districts in India that did not have close elections between female and male politicians. For three out of five district characteristics we see no significant differences. For Urban population and Female literacy rate we have a significant difference. The means for these in both samples are in line but the small SE are what drives down p-values. In any case our estimation results are produced by including these five district level variables as controls.

**Table S1.3:**

Comparing districts with and without close election.

|  | **Districts with close elections** | | **Districts without close elections** | |  |
| --- | --- | --- | --- | --- | --- |
|  | **mean** | **SE** | **mean** | **SE** | **diff P-value** |
| Urban Population | 0.274 | 0.008 | 0.227 | 0.004 | 0.000 |
|  |  |  |  |  |  |
| SC and ST Population | 0.240 | 0.005 | 0.248 | 0.004 | 0.225 |
|  |  |  |  |  |  |
| Female Population | 0.483 | 0.001 | 0.482 | 0.000 | 0.794 |
|  |  |  |  |  |  |
| Male literacy rate | 0.378 | 0.003 | 0.373 | 0.002 | 0.204 |
|  |  |  |  |  |  |
| Female literacy rate | 0.256 | 0.004 | 0.241 | 0.002 | 0.000 |
| District year Obs | 631 |  | 1499 |  |  |

*S1.4 Covariate balance*

We verify whether there is any discontinuity at the treatment threshold against the electoral margin on a range of electoral variables such as: total number of candidates, number of female candidates, number of male candidates, number of electors, female electors, male electors and votes received (total, female and male). A visual inspection of Figure S1.4 seems to indicate that they do not vary discontinuously at the RD threshold. This gives us confidence that our results do not reflect pre-existing political differences across places where women won or did not win elections.

**Figure S1.4:**

Various electoral variables plotted against margin of victory.

*S1.5 Density of vote margins*

Figure A1.5 shows that vote margin appears continuous at the discontinuity point. To confirm this, we also use the McCary density test (lower graph) to investigate the differences in the distribution density of the forcing variable around the treatment threshold of zero. With an estimated log difference of 0.0611 (p-value=0.1896) we fail to reject the null hypothesis that there is no discontinuity.

**Figure S1.5**:

****Density of margin of victory

****McCary test of discontinuity

**TABLES**

**Table: First stage regression**

|  | (1) | (2) | (3) | (4) | (5) | (6) |
| --- | --- | --- | --- | --- | --- | --- |
| Dep. variable: proportion of seats in a district won by female politicians | **Underweight** | | | | | |
|  | **0-60 months** | | | **0-24 months** | | |
| **Panel A: 2SLS regressions with no electoral margins (no polynomials)** |  |  |  |  |  |  |
| Proportion of seats in a district won by female politician | 0.902^***^ | 0.902^***^ | 0.914^***^ | 0.845^***^ | 0.845^***^ | 0.868^***^ |
| in close elections against male politician | (0.029) | (0.029) | (0.029) | (0.046) | (0.046) | (0.044) |
| Proportion of seats in a district that had close elections between | -0.305^***^ | -0.305^***^ | -0.310^***^ | -0.305^***^ | -0.306^***^ | -0.321^***^ |
| male and female candidates | (0.020) | (0.020) | (0.020) | (0.030) | (0.030) | (0.030) |
| Controls for Electoral margins | No | No | No | No | No | No |
| No individual & District controls | Yes |  |  | Yes |  |  |
| Individual controls |  | Yes |  |  | Yes |  |
| Individual and District controls |  |  | Yes |  |  | Yes |
| Cohort FE | Yes | Yes | Yes | Yes | Yes | Yes |
| District FE | Yes | Yes | Yes | Yes | Yes | Yes |
| *F stat* | *958.1* | *958.3* | *987.4* | *338.1* | *338* | *381.1* |
| *Adj. R2* | *0.854* | *0.854* | *0.854* | *0.928* | *0.928* | *0.929* |
| **Panel B: 2SLS regressions with Linear margins** |  |  |  |  |  |  |
| Proportion of seats in a district won by female politician | 0.792^***^ | 0.792^***^ | 0.796^***^ | 0.849^***^ | 0.849^***^ | 0.851^***^ |
| in close elections against male politician | (0.018) | (0.018) | (0.018) | (0.027) | (0.027) | (0.027) |
| Proportion of seats in a district that had close elections between | -0.464^***^ | -0.464^***^ | -0.467^***^ | -0.484^***^ | -0.484^***^ | -0.489^***^ |
| male and female candidates | (0.014) | (0.014) | (0.014) | (0.016) | (0.016) | (0.016) |
| Controls for Electoral margins | 2nd Order | 2nd Order | 2nd Order | 2nd Order | 2nd Order | 2nd Order |
| No individual & District controls | Yes |  |  | Yes |  |  |
| Individual controls |  | Yes |  |  | Yes |  |
| Individual and District controls |  |  | Yes |  |  | Yes |
| Cohort FE | Yes | Yes | Yes | Yes | Yes | Yes |
| District FE | Yes | Yes | Yes | Yes | Yes | Yes |
| F stat | *1914.7* | *1915.2* | *1904.1* | *992.4* | *992.7* | *1027.2* |
| Adj. R2 | *0.941* | *0.941* | *0.941* | *0.969* | *0.969* | *0.969* |
| **Panel C: 2SLS regressions with 2nd Order polynomials** |  |  |  |  |  |  |
| Proportion of seats in a district won by female politician | 0.813*** | 0.813*** | 0.817*** | 0.868*** | 0.868*** | 0.873*** |
| in close elections against male politician | (0.018) | (0.018) | (0.018) | (0.024) | (0.024) | (0.024) |
| Proportion of seats in a district that had close elections between | -0.464*** | -0.464*** | -0.466*** | -0.460*** | -0.460*** | -0.463*** |
| male and female candidates | (0.014) | (0.014) | (0.014) | (0.015) | (0.015) | (0.015) |
| Controls for Electoral margins | 2nd Order | 2nd Order | 2nd Order | 2nd Order | 2nd Order | 2nd Order |
| No individual & District controls | Yes |  |  | Yes |  |  |
| Individual controls |  | Yes |  |  | Yes |  |
| Individual and District controls |  |  | Yes |  |  | Yes |
| Cohort FE | Yes | Yes | Yes | Yes | Yes | Yes |
| District FE | Yes | Yes | Yes | Yes | Yes | Yes |
| F stat | *2087.7* | *2088.1* | *2070.2* | *1288.4* | *1287.9* | *1364.8* |
| Adj. R2 | *0.944* | *0.944* | *0.945* | *0.972* | *0.972* | *0.972* |
| **Panel D: 2SLS regressions with 3rd Order polynomials** | | | |  |  |  |
| Proportion of seats in a district won by female politician | 0.813*** | 0.813*** | 0.815*** | 0.788*** | 0.788*** | 0.796*** |
| in close elections against male politician | (0.015) | (0.015) | (0.015) | (0.020) | (0.020) | (0.020) |
| Proportion of seats in a district that had close elections between | -0.457*** | -0.457*** | -0.459*** | -0.456*** | -0.456*** | -0.458*** |
| male and female candidates | (0.012) | (0.012) | (0.012) | (0.012) | (0.012) | (0.012) |
| Controls for Electoral margins | 3rd Order | 3rd Order | 3rd Order | 3rd Order | 3rd Order | 3rd Order |
| No individual & District controls | Yes |  |  | Yes |  |  |
| Individual controls |  | Yes |  |  | Yes |  |
| Individual and District controls |  |  | Yes |  |  | Yes |
| Cohort FE | Yes | Yes | Yes | Yes | Yes | Yes |
| District FE | Yes | Yes | Yes | Yes | Yes | Yes |
| F stat | *2912.4* | 2912.6 | 2899.3 | *1430.1* | 1429.5 | 1559.5 |
| Adj. R2 | *0.958* | 0.958 | 0.958 | *0.980* | 0.980 | 0.980 |
| **Observations** | **122926** | **122926** | **122926** | **46349** | **46349** | **46349** |

Note: see note at the bottom of Table 3; Results are respectively similar when we use the outcome variable (in the second stage, see Table 3) of ‘Severely underweight age 0-60 months’ and ‘Severely underweight age 0-24 months’.

Comments on Table *First stage regression*: In panel A we use different sets of controls: individual ($X_{idt}$ and $H_{idt})$ and district ($D_{idt})$ones. In panel B we control for margins of victory or defeat with a linear polynomial, in panel C with second order polynomials and in panel D with third-order polynomials. The first stage results suggest that the instrumental variable, the proportion of seats won by women politician in a close election against male candidate, is a strong predictor for the proportion of seats won by women politician. The large F-statistics of first stage regression results appears to rule out any issue related to weak instrument.

For children under five years, column 1 to 3, results across panels do not markedly differ. Estimates in Panel D, based on the most complete specifications, show that when holding everything else constant (namely the total share of seats in close elections by district): increasing the proportion of seats won by women politician in close elections against male candidates by 10 percentage points would increase the proportion of seats won by women politicians by 8.1 percentage points. The results for children under two years are shown in columns 4 to 6. Results in panel D suggests that increasing the proportion of seats won by women politician in close election against male candidate by 10 percentage points would increase the proportion of seats in the constituencies won by women politician by 7.9 percentage points (everything else being held constant).

**Table S3.1:** Replication of the main results presented in Table 3 but based on NCHD references.

|  | **(1)** | **(2)** | **(3)** | **(4)** |
| --- | --- | --- | --- | --- |
|  | **Underweight** | | **Severely underweight** | |
|  | **0-60 months** | **0-24 months** | **0-60 months** | **0-24 months** |
| **Panel A: 2SLS regressions with no electoral margins (no polynomials)** |  |  |  |  |
| No individual & district controls: proportion of seats won by female politician | -0.387*** | -0.200 | -0.354*** | -0.162 |
|  | (0.115) | (0.229) | (0.084) | (0.163) |
| With Individual controls: proportion of seats won by female politician | -0.394*** | -0.151 | -0.353*** | -0.146 |
|  | (0.113) | (0.222) | (0.084) | (0.163) |
| With individual and district controls: proportion of seats won by female politician | -0.401*** | -0.076 | -0.364*** | -0.060 |
|  | (0.112) | (0.217) | (0.083) | (0.159) |
| Controls for Electoral margins | No | No | No | No |
| Cohort FE | yes | yes | yes | yes |
| District FE | yes | yes | yes | yes |
| **Panel B: 2SLS regressions with Linear margins** | | | |  |
| No individual & district controls: proportion of seats won by female politician | -0.500*** | -0.159 | -0.424*** | -0.115 |
|  | (0.134) | (0.243) | (0.098) | (0.174) |
| With Individual controls: proportion of seats won by female politician | -0.511*** | -0.212 | -0.424*** | -0.159 |
|  | (0.132) | (0.233) | (0.097) | (0.173) |
| With individual and district controls: proportion of seats won by female politician | -0.491*** | -0.148 | -0.423*** | -0.085 |
|  | (0.131) | (0.234) | (0.097) | (0.174) |
| Controls for Electoral margins | 1st Order | 1st Order | 1st Order | 1st Order |
| Cohort FE | yes | yes | yes | yes |
| District FE | yes | yes | yes | yes |
| **Observations** | **122926** | **46349** | **122926** | **46349** |

|  | **(1)** | **(2)** | **(3)** | **(4)** |
| --- | --- | --- | --- | --- |
|  | **Underweight** | | **Severely underweight** | |
|  | **0-60 months** | **0-24 months** | **0-60 months** | **0-24 months** |
| **Panel C: 2SLS regressions with 2nd Order polynomials** | | | |  |
| No individual & district controls: proportion of seats won by female politician | -0.480*** | -0.188 | -0.431*** | -0.158 |
|  | (0.131) | (0.243) | (0.095) | (0.174) |
| With Individual controls: proportion of seats won by female politician | -0.489*** | -0.252 | -0.430*** | -0.209 |
|  | (0.129) | (0.233) | (0.094) | (0.173) |
| With individual and district controls: proportion of seats won by female politician | -0.466*** | -0.180 | -0.429*** | -0.131 |
|  | (0.128) | (0.233) | (0.094) | (0.173) |
| Controls for Electoral margins | 2nd Order | 2nd Order | 2nd Order | 2nd Order |
| Cohort FE | yes | yes | yes | yes |
| District FE | yes | yes | yes | yes |
| **Panel D: 2SLS regressions with 3rd Order polynomials** | | | |  |
| No individual & district controls: proportion of seats won by female politician | -0.377*** | -0.178 | -0.368*** | -0.368*** |
|  | (0.131) | (0.268) | (0.096) | (0.096) |
| With Individual controls: proportion of seats won by female politician | -0.387*** | -0.257 | -0.367*** | -0.214 |
|  | (0.129) | (0.258) | (0.095) | (0.191) |
| With individual and district controls: proportion of seats won by female politician | -0.362*** | -0.179 | -0.367*** | -0.129 |
|  | (0.129) | (0.257) | (0.095) | (0.191) |
| Controls for Electoral margins | 3rd Order | 3rd Order | 3rd Order | 3rd Order |
| Cohort FE | yes | yes | yes | yes |
| District FE | yes | yes | yes | yes |
| **Observations** | **122926** | **46349** | **122926** | **46349** |

Note: Robust standard errors clustered at districts primary sampling unit reported in parentheses. All regression equations include cohorts and district fixed effects. Individual controls include child gender, child age in months, mother age at childbirth, mothers' education level, caste (gen, SC, ST), religion (Hindu, Muslim), live in rural areas, and Socio-economics status index. District level controls include female population, urban population, SC/ST population, and literacy rate. Also, all 2SLS regressions are controlled for the proportion of constituencies in a district that had total close elections between women and men. ^*^ *p* < 0.1, ^**^ *p* < 0.05, ^***^ *p* < 0.01.

**Table S3.2:** Replications of the results from Table 3 for the sample of districts with at-least one election between woman-man

|  | **(1)** | **(2)** | **(3)** | **(4)** |
| --- | --- | --- | --- | --- |
|  | **Underweight** | | **Severely underweight** | |
|  | **0-60 months** | **0-24 months** | **0-60 months** | **0-24 months** |
| **Panel A: 2SLS regressions with no electoral margins (no polynomials)** | | | |  |
| No individual & district controls: proportion of seats won by female politician | -0.234^*^ | -0.346 | -0.091 | -0.306 |
|  | (0.121) | (0.254) | (0.099) | (0.213) |
| With Individual controls: proportion of seats won by female politician | -0.256^**^ | -0.264 | -0.099 | -0.267 |
|  | (0.119) | (0.249) | (0.099) | (0.212) |
| With individual and district controls: proportion of seats won by female politician | -0.321^***^ | -0.21 | -0.158 | -0.252 |
|  | (0.115) | (0.239) | (0.096) | (0.204) |
| Controls for Electoral margins | No | No | No | No |
| Cohort FE | yes | yes | yes | yes |
| District FE | yes | yes | yes | yes |
| **Panel B: 2SLS regressions with Linear margins** |  |  |  |  |
| No individual & district controls: proportion of seats won by female politician | -0.330^**^ | -0.212 | -0.125 | -0.223 |
|  | (0.140) | (0.265) | (0.115) | (0.220) |
| With Individual controls: proportion of seats won by female politician | -0.362^***^ | -0.22 | -0.138 | -0.236 |
|  | (0.138) | (0.258) | (0.115) | (0.218) |
| With individual and district controls: proportion of seats won by female politician | -0.363^***^ | -0.152 | -0.158 | -0.215 |
|  | (0.132) | (0.255) | (0.111) | (0.216) |
| Controls for Electoral margins | 1st Order | 1st Order | 1st Order | 1st Order |
| Cohort FE | yes | yes | yes | yes |
| District FE | yes | yes | yes | yes |
| **Panel C: 2SLS regressions with 2nd Order polynomials** |  |  |  |  |
| No individual & district controls: proportion of seats won by female politician | -0.324^**^ | -0.186 | -0.129 | -0.228 |
|  | (0.135) | (0.264) | (0.111) | (0.221) |
| With Individual controls: proportion of seats won by female politician | -0.354^***^ | -0.202 | -0.142 | -0.246 |
|  | (0.134) | (0.258) | (0.111) | (0.220) |
| With individual and district controls: proportion of seats won by female politician | -0.352^***^ | -0.143 | -0.158 | -0.236 |
|  | (0.128) | (0.255) | (0.107) | (0.219) |
| Controls for Electoral margins | 2nd Order | 2nd Order | 2nd Order | 2nd Order |
| Cohort FE | yes | yes | yes | yes |
| District FE | yes | yes | yes | yes |
| **Panel D: 2SLS regressions with 3rd Order polynomials** |  |  |  |  |
| No individual & district controls: proportion of seats won by female politician | -0.231 | -0.173 | -0.049 | -0.239 |
|  | (0.146) | (0.299) | (0.120) | (0.249) |
| With Individual controls: proportion of seats won by female politician | -0.267^*^ | -0.198 | -0.065 | -0.263 |
|  | (0.144) | (0.292) | (0.12) | (0.248) |
| With individual and district controls: proportion of seats won by female politician | -0.262^*^ | -0.127 | -0.081 | -0.253 |
|  | (0.140) | (0.288) | (0.117) | (0.246) |
| Controls for Electoral margins | 3rd Order | 3rd Order | 3rd Order | 3rd Order |
| Cohort FE | yes | yes | yes | yes |
| District FE | yes | yes | yes | yes |
| **Observations** | 70722 | 28264 | 70722 | 28264 |

Note: see note at the bottom of Table S3.1.

**Table S4:** Robustness checks using various election thresholds; using dummy dependent variables (underweight and severely underweight).

| *Estimated coefficients* | (1) | (2) | (3) | (4) | (5) | (6) | (7) | (8) | (9) | **(10)** | **(11)** | **(12)** |
| --- | --- | --- | --- | --- | --- | --- | --- | --- | --- | --- | --- | --- |
| *for Proportion of seats won* | **Underweight** | | | | | | **Severely underweight** | | | | | |
| *by female politicians* | **0-60 months** | | | **0-24 months** | | | **0-60 months** | | | **0-24 months** | | |
| **Panel A: 2SLS regressions with no electoral margins (no polynomials)** | | | | | | | | | |  |  |  |
| *Close election cut-off 2%* | -0.364^*^ | -0.378^**^ | -0.408^**^ | -0.549^*^ | -0.500^*^ | -0.519^*^ | -0.379^**^ | -0.386^**^ | -0.428^***^ | -0.623^**^ | -0.601^**^ | -0.611^***^ |
|  | (0.193) | (0.190) | (0.186) | (0.286) | (0.279) | (0.274) | (0.161) | (0.160) | (0.157) | (0.243) | (0.241) | (0.237) |
| *Close election cut-off 2.5%* | -0.304^**^ | -0.325^**^ | -0.357^**^ | -0.633^**^ | -0.563^**^ | -0.556^**^ | -0.274^**^ | -0.284^**^ | -0.324^***^ | -0.546^**^ | -0.512^**^ | -0.503^**^ |
|  | (0.149) | (0.147) | (0.144) | (0.260) | (0.255) | (0.249) | (0.123) | (0.122) | (0.120) | (0.218) | (0.217) | (0.212) |
| *Close election cut-off 3%* | -0.275^**^ | -0.273^**^ | -0.303^**^ | -0.471^*^ | -0.357 | -0.334 | -0.227^**^ | -0.225^**^ | -0.258^**^ | -0.287 | -0.227 | -0.208 |
|  | (0.129) | (0.127) | (0.125) | (0.278) | (0.274) | (0.268) | (0.105) | (0.105) | (0.103) | (0.231) | (0.230) | (0.226) |
| *Close election cut-off 4%* | -0.348^***^ | -0.365^***^ | -0.374^***^ | -0.609^***^ | -0.566^***^ | -0.509^**^ | -0.307^***^ | -0.314^***^ | -0.331^***^ | -0.410^**^ | -0.391^**^ | -0.348^**^ |
|  | (0.111) | (0.109) | (0.108) | (0.217) | (0.213) | (0.209) | (0.089) | (0.089) | (0.088) | (0.181) | (0.180) | (0.177) |
| *Close election cut-off 4.5%* | -0.418^***^ | -0.427^***^ | -0.415^***^ | -0.633^***^ | -0.592^***^ | -0.562^***^ | -0.339^***^ | -0.344^***^ | -0.347^***^ | -0.401^**^ | -0.384^**^ | -0.361^**^ |
|  | (0.105) | (0.103) | (0.102) | (0.206) | (0.202) | (0.2) | (0.084) | (0.084) | (0.083) | (0.171) | (0.170) | (0.169) |
| *Close election cut-off 5%* | -0.406^***^ | -0.425^***^ | -0.422^***^ | -0.673^***^ | -0.650^***^ | -0.619^***^ | -0.322^***^ | -0.331^***^ | -0.341^***^ | -0.371^**^ | -0.365^**^ | -0.344^**^ |
|  | (0.105) | (0.103) | (0.102) | (0.216) | (0.210) | (0.208) | (0.084) | (0.084) | (0.083) | (0.173) | (0.172) | (0.170) |
| *Close election cut-off 5.5%* | -0.374^***^ | -0.387^***^ | -0.377^***^ | -0.521^***^ | -0.481^**^ | -0.436^**^ | -0.259^***^ | -0.265^***^ | -0.270^***^ | -0.228 | -0.21 | -0.183 |
|  | (0.099) | (0.097) | (0.096) | (0.200) | (0.194) | (0.192) | -0.079 | (0.078) | (0.077) | (0.160) | (0.158) | (0.157) |
| *Close election cut-off 6%* | -0.369^***^ | -0.382^***^ | -0.371^***^ | -0.519^***^ | -0.479^**^ | -0.440^**^ | -0.246^***^ | -0.252^***^ | -0.256^***^ | -0.216 | -0.198 | -0.174 |
|  | (0.098) | (0.096) | (0.095) | (0.195) | (0.190) | (0.188) | (0.078) | (0.077) | (0.077) | (0.157) | (0.155) | (0.154) |
| **Panel B: 2SLS regressions with Linear margins** | | | | | | | | | |  |  |  |
| *Close election cut-off 2%* | -0.352^*^ | -0.367^**^ | -0.360^**^ | -0.41 | -0.414 | -0.466 | -0.353^**^ | -0.360^**^ | -0.378^**^ | -0.535^**^ | -0.546^**^ | -0.582^**^ |
|  | (0.189) | (0.186) | (0.184) | (0.304) | (0.294) | (0.294) | (0.158) | (0.156) | (0.154) | (0.256) | (0.252) | (0.253) |
| *Close election cut-off 2.5%* | -0.349^**^ | -0.375^**^ | -0.367^**^ | -0.487^*^ | -0.481^*^ | -0.501^*^ | -0.284^**^ | -0.297^**^ | -0.314^**^ | -0.447^*^ | -0.452^*^ | -0.464^*^ |
|  | (0.164) | (0.161) | (0.159) | (0.293) | (0.285) | (0.284) | (0.135) | (0.134) | (0.133) | (0.242) | (0.240) | (0.240) |
| *Close election cut-off 3%* | -0.326^**^ | -0.325^**^ | -0.323^**^ | -0.418 | -0.38 | -0.376 | -0.239^**^ | -0.237^**^ | -0.251^**^ | -0.244 | -0.23 | -0.223 |
|  | (0.144) | (0.142) | (0.141) | (0.299) | (0.293) | (0.293) | (0.118) | (0.117) | (0.117) | (0.247) | (0.245) | (0.246) |
| *Close election cut-off 4%* | -0.464^***^ | -0.488^***^ | -0.465^***^ | -0.651^***^ | -0.658^***^ | -0.612^***^ | -0.359^***^ | -0.369^***^ | -0.366^***^ | -0.435^**^ | -0.448^**^ | -0.415^**^ |
|  | (0.126) | (0.124) | (0.123) | (0.238) | (0.232) | (0.232) | (0.101) | (0.100) | (0.099) | (0.196) | (0.195) | (0.196) |
| *Close election cut-off 4.5%* | -0.539^***^ | -0.551^***^ | -0.513^***^ | -0.727^***^ | -0.723^***^ | -0.701^***^ | -0.393^***^ | -0.399^***^ | -0.386^***^ | -0.449^**^ | -0.456^**^ | -0.439^**^ |
|  | (0.121) | (0.119) | (0.119) | (0.234) | (0.228) | (0.229) | (0.097) | (0.096) | (0.096) | (0.193) | (0.191) | (0.192) |
| *Close election cut-off 5%* | -0.494^***^ | -0.513^***^ | -0.489^***^ | -0.760^***^ | -0.771^***^ | -0.755^***^ | -0.356^***^ | -0.365^***^ | -0.362^***^ | -0.404^**^ | -0.419^**^ | -0.407^**^ |
|  | (0.121) | (0.119) | (0.119) | (0.234) | (0.227) | (0.228) | (0.097) | (0.096) | (0.096) | (0.185) | (0.183) | (0.184) |
| *Close election cut-off 5.5%* | -0.484^***^ | -0.496^***^ | -0.467^***^ | -0.609^***^ | -0.593^***^ | -0.556^***^ | -0.304^***^ | -0.309^***^ | -0.304^***^ | -0.269 | -0.266 | -0.244 |
|  | (0.115) | (0.113) | (0.113) | (0.216) | (0.209) | (0.210) | (0.091) | (0.091) | (0.090) | (0.171) | (0.169) | (0.170) |
| *Close election cut-off 6%* | -0.480^***^ | -0.491^***^ | -0.461^***^ | -0.621^***^ | -0.604^***^ | -0.571^***^ | -0.292^***^ | -0.298^***^ | -0.290^***^ | -0.263 | -0.259 | -0.239 |
|  | (0.115) | (0.113) | (0.113) | (0.214) | (0.207) | (0.208) | (0.091) | (0.090) | (0.090) | (0.170) | (0.168) | (0.169) |
| **Panel C: 2SLS regressions with 3rd Order polynomials** | | | | | | | | | |  |  |  |
| *Close election cut-off 2%* | -0.297^*^ | -0.314^*^ | -0.300^*^ | -0.51 | -0.528^*^ | -0.580^*^ | -0.298^**^ | -0.307^**^ | -0.322^**^ | -0.584^**^ | -0.603^**^ | -0.641^**^ |
|  | (0.175) | (0.171) | (0.170) | (0.320) | (0.311) | (0.310) | (0.145) | (0.143) | (0.143) | (0.271) | (0.267) | (0.267) |
| *Close election cut-off 2.5%* | -0.289^*^ | -0.315^**^ | -0.293^**^ | -0.494 | -0.524^*^ | -0.539^*^ | -0.227^*^ | -0.241^*^ | -0.250^**^ | -0.447^*^ | -0.452^*^ | -0.464^*^ |
|  | (0.152) | (0.149) | (0.148) | (0.309) | (-0.300) | (0.298) | (0.125) | (0.124) | (0.123) | (0.242) | (0.240) | (0.240) |
| *Close election cut-off 3%* | -0.272^*^ | -0.271^**^ | -0.259^*^ | -0.422 | -0.394 | -0.370 | -0.195^*^ | -0.193^*^ | -0.203^*^ | -0.299 | -0.291 | -0.275 |
|  | (0.139) | (0.137) | (0.136) | (0.317) | (0.312) | (0.309) | (0.113) | (0.113) | (0.112) | (0.263) | (0.261) | (0.261) |
| *Close election cut-off 4%* | -0.414^***^ | -0.439^***^ | -0.405^***^ | -0.775^***^ | -0.793^***^ | -0.732^***^ | -0.326^***^ | -0.337^***^ | -0.331^***^ | -0.527^**^ | -0.547^**^ | -0.509^**^ |
|  | (0.127) | (0.125) | (0.124) | (0.272) | (0.267) | (0.265) | (0.101) | (0.100) | (0.100) | (0.225) | (0.224) | (0.223) |
| *Close election cut-off 4.5%* | -0.526^***^ | -0.539^***^ | -0.487^***^ | -0.854^***^ | -0.867^***^ | -0.830^***^ | -0.385^***^ | -0.391^***^ | -0.374^***^ | -0.552^**^ | -0.571^**^ | -0.545^**^ |
|  | (0.126) | (0.124) | (0.124) | (0.270) | (0.265) | (0.263) | (0.100) | (0.099) | (0.099) | (0.225) | (0.223) | (0.223) |
| *Close election cut-off 5%* | -0.505^***^ | -0.528^***^ | -0.486^***^ | -0.935^***^ | -0.971^***^ | -0.949^***^ | -0.363^***^ | -0.375^***^ | -0.365^***^ | -0.502^**^ | -0.534^**^ | -0.522^**^ |
|  | (0.129) | (0.127) | (0.126) | (0.279) | (0.273) | (0.272) | (0.103) | (0.102) | (0.101) | (0.225) | (0.223) | (0.223) |
| *Close election cut-off 5.5%* | -0.547^***^ | -0.565^***^ | -0.512^***^ | -0.778^***^ | -0.788^***^ | -0.744^***^ | -0.343^***^ | -0.352^***^ | -0.335^***^ | -0.338 | -0.352^*^ | -0.331 |
|  | (0.126) | (0.124) | (0.123) | (0.263) | (0.257) | (0.256) | (0.099) | (0.098) | (0.098) | (0.213) | (0.211) | (0.211) |
| *Close election cut-off 6%* | -0.537^***^ | -0.555^***^ | -0.499^***^ | -0.827^***^ | -0.839^***^ | -0.799^***^ | -0.327^***^ | -0.336^***^ | -0.316^***^ | -0.352 | -0.365^*^ | -0.346 |
|  | (0.125) | (0.123) | (0.123) | (0.267) | (0.262) | (0.260) | (0.099) | (0.098) | (0.098) | (0.218) | (0.217) | (0.216) |
| No individual & district controls | Yes |  |  | Yes |  |  | Yes |  |  | Yes |  |  |
| With Individual controls |  | Yes |  |  | Yes |  |  | Yes |  |  | Yes |  |
| With individual and district controls | |  | Yes |  |  | Yes |  |  | Yes |  |  | Yes |
| Cohort FE | Yes | Yes | Yes | Yes | Yes | Yes | Yes | Yes | Yes | Yes | Yes | Yes |
| District FE | Yes | Yes | Yes | Yes | Yes | Yes | Yes | Yes | Yes | Yes | Yes | Yes |
| Observations | 122926 | 122926 | 122926 | 46349 | 46349 | 46349 | 122926 | 122926 | 122926 | 46349 | 46349 | 46349 |

Note: See note for Table S3.1.

**Table S5:** Robustness checks using various election thresholds; using weight-for-age (WAZ) z-scores.

|  | (1) | (2) | (3) | (4) | (5) | (6) | (7) | (8) | (9) | (10) | (11) | (12) |
| --- | --- | --- | --- | --- | --- | --- | --- | --- | --- | --- | --- | --- |
|  | **WAZ (as per WHO growth reference 2006)** | | | | | | **WAZ (as per NCHD growth reference in DLHS)** | | | | | |
|  | **aged 0 to 60 months** | | | **aged 0 to 24 months** | | | **aged 0 to 60 months** | | | **aged 0 to 24 months** | | |
| **Panel A: 2SLS regressions with no electoral margins (no polynomials)** |  |  |  |  |  |  |  |  |  |  |  |  |
| *Close election cut-off 2%*: proportion of seats won by female politician | 0.828 | 0.881 | 1.104^*^ | 2.046^*^ | 1.896^*^ | 1.916^*^ | 1.406^**^ | 1.441^**^ | 1.555^***^ | 1.792^*^ | 1.634^*^ | 1.525 |
|  | (0.626) | (0.611) | (0.594) | (1.095) | (1.063) | (1.042) | (0.598) | (0.584) | (0.566) | (1.029) | (0.982) | (0.963) |
| *Close election cut-off 2.5%*: proportion of seats won by female politician | 0.694 | 0.778^*^ | 1.006^**^ | 2.120^**^ | 1.856^*^ | 1.829^*^ | 1.186^***^ | 1.256^***^ | 1.397^***^ | 1.859^**^ | 1.514^*^ | 1.369 |
|  | (0.478) | (0.467) | (0.454) | (0.983) | (0.959) | (0.938) | (0.457) | (0.446) | (0.433) | (0.925) | (0.885) | (0.865) |
| *Close election cut-off 3%*: proportion of seats won by female politician | 0.371 | 0.36 | 0.563 | 1.19 | 0.764 | 0.704 | 0.898^**^ | 0.874^**^ | 1.034^***^ | 1.09 | 0.572 | 0.372 |
|  | (0.407) | (0.399) | (0.390) | (1.03) | (1.011) | (0.987) | (0.388) | (0.380) | (0.372) | (0.966) | (0.930) | (0.908) |
| *Close election cuttoff 4%*: proportion of seats won by female politician | 0.816^**^ | 0.900^***^ | 1.018^***^ | 1.853^**^ | 1.739^**^ | 1.602^**^ | 1.315^***^ | 1.398^***^ | 1.475^***^ | 1.389^*^ | 1.317^*^ | 1.115 |
|  | (0.350) | (0.342) | (0.335) | (0.795) | (0.776) | (0.763) | (0.341) | (0.333) | (0.326) | (0.747) | (0.711) | (0.700) |
| *Close election cut-off 4.5%*: proportion of seats won by female politician | 0.981^***^ | 1.029^***^ | 1.090^***^ | 2.166^***^ | 2.046^***^ | 1.953^***^ | 1.314^***^ | 1.359^***^ | 1.408^***^ | 1.795^**^ | 1.694^**^ | 1.547^**^ |
|  | (0.327) | (0.320) | (0.314) | (0.762) | (0.744) | (0.738) | (0.320) | (0.313) | (0.306) | (0.716) | (0.682) | (0.676) |
| *Close election cut-off 5%*: proportion of seats won by female politician | 1.102^***^ | 1.187^***^ | 1.278^***^ | 2.114^***^ | 2.101^***^ | 2.004^***^ | 1.355^***^ | 1.438^***^ | 1.522^***^ | 1.743^**^ | 1.831^***^ | 1.680^**^ |
|  | (0.328) | (0.320) | (0.315) | (0.785) | (0.758) | (0.751) | (0.320) | (0.313) | (0.307) | (0.738) | (0.694) | (0.687) |
| *Close election cut-off 5.5%*: proportion of seats won by female politician | 0.933^***^ | 0.996^***^ | 1.059^***^ | 1.523^**^ | 1.434^**^ | 1.283^*^ | 1.067^***^ | 1.131^***^ | 1.189^***^ | 1.161^*^ | 1.154^*^ | 0.976 |
|  | (0.310) | (0.303) | (0.298) | (0.739) | (0.709) | (0.704) | (0.304) | (0.297) | (0.291) | (0.697) | (0.651) | (0.645) |
| **Panel B: 2SLS regressions with Linear margins** |  |  |  |  |  |  |  |  |  |  |  |  |
| *Close election cut-off 2%*: proportion of seats won by female politician | 0.852 | 0.918 | 0.968^*^ | 1.473 | 1.568 | 1.676 | 1.551^***^ | 1.603^***^ | 1.552^***^ | 1.227 | 1.421 | 1.396 |
|  | (0.610) | (0.595) | (0.585) | (1.196) | (1.153) | (1.152) | (0.587) | (0.570) | (0.561) | (1.134) | (1.073) | (1.073) |
| *Close election cut-off 2.5%*: proportion of seats won by female politician | 0.795 | 0.909^*^ | 0.985^*^ | 1.458 | 1.511 | 1.55 | 1.422^***^ | 1.525^***^ | 1.515^***^ | 1.152 | 1.291 | 1.202 |
|  | (0.523) | (0.510) | (0.503) | (1.14) | (1.102) | (1.100) | (0.503) | (0.489) | (0.482) | (1.08) | (1.023) | (1.021) |
| *Close election cut-off 3%*: proportion of seats won by female politician | 0.444 | 0.446 | 0.529 | 0.849 | 0.781 | 0.751 | 1.120^**^ | 1.111^***^ | 1.155^***^ | 0.749 | 0.756 | 0.583 |
|  | (0.454) | (0.445) | (0.440) | (1.155) | (1.127) | (1.126) | (0.435) | (0.425) | (0.421) | (1.097) | (1.047) | (1.047) |
| *Close election cuttoff 4%*: proportion of seats won by female politician | 1.167^***^ | 1.289^***^ | 1.277^***^ | 2.051^**^ | 2.175^**^ | 2.039^**^ | 1.899^***^ | 2.025^***^ | 1.972^***^ | 1.606^*^ | 1.887^**^ | 1.684^**^ |
|  | (0.398) | (0.389) | (0.385) | (0.915) | (0.885) | (0.884) | (0.391) | (0.382) | (0.377) | (0.867) | (0.816) | (0.816) |
| *Close election cut-off 4.5%*: proportion of seats won by female politician | 1.291^***^ | 1.358^***^ | 1.310^***^ | 2.509^***^ | 2.566^***^ | 2.470^***^ | 1.845^***^ | 1.912^***^ | 1.853^***^ | 2.152^**^ | 2.315^***^ | 2.168^***^ |
|  | (0.378) | (0.370) | (0.367) | (0.899) | (0.871) | (0.873) | (0.372) | (0.363) | (0.359) | (0.852) | (0.804) | (0.806) |
| *Close election cut-off 5%*: proportion of seats won by female politician | 1.298^***^ | 1.391^***^ | 1.403^***^ | 2.440^***^ | 2.583^***^ | 2.494^***^ | 1.736^***^ | 1.829^***^ | 1.833^***^ | 2.090^**^ | 2.403^***^ | 2.265^***^ |
|  | (0.381) | (0.372) | (0.369) | (0.877) | (0.841) | (0.845) | (0.374) | (0.364) | (0.360) | (0.830) | (0.774) | (0.777) |
| *Close election cut-off 5.5%*: proportion of seats won by female politician | 1.179^***^ | 1.246^***^ | 1.236^***^ | 1.831^**^ | 1.851^**^ | 1.689^**^ | 1.457^***^ | 1.525^***^ | 1.506^***^ | 1.475^*^ | 1.624^**^ | 1.440^**^ |
|  | (0.364) | (0.355) | -0.352 | (0.815) | (0.779) | (0.782) | (0.358) | (0.349) | (0.345) | (0.772) | (0.716) | (0.718) |

**Panel C: 2SLS regressions with 3rd Order polynomials**

| *Close election cut-off 2%*: proportion of seats won by female politician | 0.688 | 0.758 | 0.799 | 1.81 | 2.007 | 2.126^*^ | 1.254^**^ | 1.309^**^ | 1.266^**^ | 1.402 | 1.794 | 1.759 |
| --- | --- | --- | --- | --- | --- | --- | --- | --- | --- | --- | --- | --- |
|  | (0.561) | (0.547) | (0.543) | (1.265) | (1.223) | (1.217) | (0.537) | (0.522) | (0.519) | (1.204) | (1.142) | (1.137) |
| *Close election cut-off 2.5%*: proportion of seats won by female politician | 0.608 | 0.719 | 0.77 | 1.539 | 1.798 | 1.84 | 1.096^**^ | 1.194^***^ | 1.180^***^ | 1.163 | 1.643 | 1.535 |
|  | (0.485) | (0.473) | (0.469) | (1.214) | (1.173) | (1.162) | (0.464) | (0.451) | (0.448) | (1.162) | (1.094) | (1.086) |
| *Close election cut-off 3%*: proportion of seats won by female politician | 0.293 | 0.292 | 0.369 | 0.881 | 0.911 | 0.853 | 0.856^**^ | 0.843^**^ | 0.892^**^ | 0.717 | 0.931 | 0.706 |
|  | (0.437) | (0.428) | (0.425) | (1.237) | (1.208) | (1.198) | (0.417) | (0.408) | (0.406) | (1.184) | (1.128) | (1.120) |
| *Close election cuttoff 4%*: proportion of seats won by female politician | 1.049^***^ | 1.175^***^ | 1.153^***^ | 2.405^**^ | 2.618^**^ | 2.449^**^ | 1.738^***^ | 1.866^***^ | 1.807^***^ | 1.793^*^ | 2.240^**^ | 1.984^**^ |
|  | (0.400) | (0.391) | (0.388) | (1.048) | (1.020) | (1.011) | (0.391) | (0.383) | (0.380) | (0.998) | (0.946) | (0.940) |
| *Close election cut-off 4.5%*: proportion of seats won by female politician | 1.280^***^ | 1.349^***^ | 1.287^***^ | 2.998^***^ | 3.170^***^ | 3.047^***^ | 1.803^***^ | 1.870^***^ | 1.797^***^ | 2.491^**^ | 2.857^***^ | 2.663^***^ |
|  | (0.394) | (0.386) | (0.383) | (1.044) | (1.016) | (1.009) | (0.386) | (0.377) | (0.375) | (0.998) | (0.945) | (0.939) |
| *Close election cut-off 5%*: proportion of seats won by female politician | 1.352^***^ | 1.461^***^ | 1.439^***^ | 3.040^***^ | 3.336^***^ | 3.240^***^ | 1.780^***^ | 1.885^***^ | 1.855^***^ | 2.537^**^ | 3.106^***^ | 2.934^***^ |
|  | (0.406) | (0.397) | (0.395) | (1.05) | (1.019) | (1.013) | (0.396) | (0.387) | (0.385) | (1.001) | (0.946) | (0.941) |
| *Close election cut-off 5.5%*: proportion of seats won by female politician | 1.377^***^ | 1.467^***^ | 1.396^***^ | 2.361^**^ | 2.529^***^ | 2.347^**^ | 1.651^***^ | 1.739^***^ | 1.656^***^ | 1.817^*^ | 2.205^**^ | 1.981^**^ |
|  | (0.397) | (0.388) | (0.386) | (0.991) | (0.961) | (0.955) | (0.389) | (0.380) | (0.377) | (0.944) | (0.890) | (0.886) |
| Controls for Electoral margins | 3rd Order | 3rd Order | 3rd Order | 3rd Order | 3rd Order | 3rd Order | 3rd Order | 3rd Order | 3rd Order | 3rd Order | 3rd Order | 3rd Order |
| No individual & district controls | yes |  |  | yes |  |  | yes |  |  | yes |  |  |
| With Individual controls |  | yes |  |  | yes |  |  | yes |  |  | yes |  |
| With individual and district controls |  |  | yes |  |  | yes |  |  | yes |  |  | yes |
| Cohort FE | Yes | Yes | Yes | Yes | Yes | Yes | Yes | Yes | Yes | Yes | Yes | Yes |
| District FE | Yes | Yes | Yes | Yes | Yes | Yes | Yes | Yes | Yes | Yes | Yes | Yes |
| Observations | 122926 | 122926 | 122926 | 46349 | 46349 | 46349 | 122926 | 122926 | 122926 | 46349 | 46349 | 46349 |

Note: same as for Table S3.1.

**Table S6:** Results based on a 3-year moving average for women political representation (election threshold of 3.5%)

|  | **(1)** | **(2)** | **(3)** | **(4)** |
| --- | --- | --- | --- | --- |
|  | **Underweight** | | **Severely underweight** | |
|  | **0-60 months** | **0-24 months** | **0-60 months** | **0-24 months** |
| **Panel A: 2SLS regressions with no electoral margins (no polynomials)** |  |  |  |  |
| No individual & district controls: proportion of seats won by female politician | -0.296^***^ | -0.469** | -0.217^**^ | -0.292 |
|  | (0.112) | (0.233) | (0.091) | (0.195) |
| With Individual controls: proportion of seats won by female politician | -0.306^***^ | -0.424* | -0.221^**^ | -0.271 |
|  | (0.111) | (0.230) | (0.091) | (0.195) |
| With individual and district controls: proportion of seats won by female politician | -0.328^***^ | -0.374* | -0.249^***^ | -0.237 |
|  | (0.109) | (0.224) | (0.089) | (0.191) |
| Controls for Electoral margins | No | No | No | No |
| Cohort FE | Yes | Yes | Yes | Yes |
| District FE | Yes | Yes | Yes | Yes |
| **Panel B: 2SLS regressions with Linear margins** |  |  |  |  |
| No individual & district controls: proportion of seats won by female politician | -0.385^***^ | -0.422* | -0.227^**^ | -0.192 |
|  | (0.130) | (0.246) | (0.106) | (0.207) |
| With Individual controls: proportion of seats won by female politician | -0.396^***^ | -0.394 | -0.231^**^ | -0.182 |
|  | (0.128) | (0.241) | (0.105) | (0.206) |
| With individual and district controls: proportion of seats won by female politician | -0.396^***^ | -0.387 | -0.250^**^ | -0.178 |
|  | (0.127) | (0.242) | (0.104) | (0.207) |
| Controls for Electoral margins | 1st Order | 1st Order | 1st Order | 1st Order |
| Cohort FE | Yes | Yes | Yes | Yes |
| District FE | Yes | Yes | Yes | Yes |
| **Panel C: 2SLS regressions with 2nd Order polynomials** |  |  |  |  |
| No individual & district controls: proportion of seats won by female politician | -0.310^**^ | -0.361 | -0.195^*^ | -0.187 |
|  | (0.126) | (0.242) | (0.102) | (0.206) |
| With Individual controls: proportion of seats won by female politician | -0.320^***^ | -0.345 | -0.198^*^ | -0.184 |
|  | (0.124) | (0.237) | (0.102) | (0.204) |
| With individual and district controls: proportion of seats won by female politician | -0.321^***^ | -0.339 | -0.217^**^ | -0.179 |
|  | (0.123) | (0.237) | (0.101) | (0.206) |
| Controls for Electoral margins | 2nd Order | 2nd Order | 2nd Order | 2nd Order |
| Cohort FE | Yes | Yes | Yes | Yes |
| District FE | Yes | Yes | Yes | Yes |
| **Panel D: 2SLS regressions with 3rd Order polynomials** |  |  |  |  |
| No individual & district controls: proportion of seats won by female politician | -0.239* | -0.436 | -0.141 | -0.206 |
|  | (0.129) | (0.268) | (0.105) | (0.227) |
| With Individual controls: proportion of seats won by female politician | -0.249* | -0.416 | -0.144 | -0.201 |
|  | (0.127) | (0.263) | (0.105) | (0.226) |
| With individual and district controls: proportion of seats won by female politician | -0.242* | -0.409 | -0.160 | -0.193 |
|  | (0.127) | (0.262) | (0.104) | (0.226) |
| Controls for Electoral margins | 3rd Order | 3rd Order | 3rd Order | 3rd Order |
| Cohort FE | yes | yes | yes | yes |
| District FE | yes | yes | yes | yes |
| **Observations** | **122926** | **46349** | **122926** | **46349** |

Note: see note for Table S3.1.

**Table S7:** Robustness checks: Using lags and leads for women political representation.

| **Coeff. For Female representation** | | | | | |
| --- | --- | --- | --- | --- | --- |
| **underweight** | | | | | |
| **Panel A: 0-24 months** | | | | | |
| **Linear** | **3rd Order** |  | **Linear** | **3rd Order** |  |
| **t-3** | | **Obs** | **t+1** | | **Obs** |
| 0.06 | 0.064 | 46290 | -0.324 | -0.443^*^ | 45379 |
| (0.254) | (0.281) |  | (0.225) | (0.256) |  |
| **t-2** | |  | **t+2** | |  |
| -0.871^***^ | -0.826^**^ | 46279 | -0.112 | -0.15 | 44078 |
| (0.317) | (0.337) |  | (0.282) | (0.289) |  |
| **t-1** | |  | **t+3** | |  |
| 0.086 | 0.099 | 46221 | 0.288 | 0.501 | 42439 |
| (0.281) | (0.261) |  | (0.308) | (0.383) |  |
| **t** | |  | **t+4** | |  |
| -0.428^*^ | -0.475^*^ | 46221 | 0.347 | 0.222 | 39580 |
| (0.243) | (0.266) |  | (0.341) | (0.323) |  |
|  | |  | **t+5** | |  |
|  |  |  | 0.553 | 0.448 | 36377 |
|  |  |  | (0.349) | (0.361) |  |

Note: For all estimations above R^2^ varies between 0.117 and 0.122

| **Coeff. For Female representation** | | | | | |
| --- | --- | --- | --- | --- | --- |
| **underweight** | | | | | |
| **Panel B: 0-60 months** | | | | | |
| **Linear** | **3rd Order** |  | **Linear** | **3rd Order** |  |
| **t-3** | | **Obs** | **t+1** | | **Obs** |
| 0.196 | 0.164 | 122100 | -0.154 | -0.12 | 121544 |
| (0.126) | (0.128) |  | (0.104) | (0.108) |  |
| **t-2** | |  | **t+2** | |  |
| -0.201 | -0.208 | 122091 | -0.167* | -0.158 | 119863 |
| (0.124) | (0.128) |  | (0.092) | (0.1) |  |
| **t-1** | |  | **t+3** | |  |
| -0.166 | -0.096 | 122216 | -0.099 | -0.09 | 117373 |
| (0.133) | (0.131) |  | (0.102) | (0.112) |  |
| **t** | |  | **t+4** | |  |
| -0.375^***^ | -0.303^**^ | 122239 | -0.106 | -0.096 | 113043 |
| (0.129) | (0.127) |  | (0.119) | (0.123) |  |
|  | |  | **t+5** | |  |
|  |  |  | -0.075 | -0.116 | 107064 |
|  |  |  | (0.12) | (0.122) |  |

Note: For all estimations above R^2^ varies between 0.1 and 0.108.

**Table S8:** Robustness checks for results presented in Table 3: including mothers Fixed Effects.

|  | (1) | (2) | (3) | (4) | (5) | (6) | (7) | (8) | (9) | (10) | (11) | (12) |
| --- | --- | --- | --- | --- | --- | --- | --- | --- | --- | --- | --- | --- |
|  | **Underweight** | | | | | | **Severely underweight** | | | | | |
|  | **0-60 months** | | | **0-24 months** | | | **0-60 months** | | | **0-24 months** | | |
| **Panel A: 2SLS regressions with Linear margins** | | | | | | | | | |  |  |  |
| Proportion of seats won | -0.499** | -0.522** | -0.534*** | 0.990 | 0.870 | 0.668 | -0.227 | -0.242 | -0.257 | 0.614 | 0.574 | 0.493 |
| by female politician | (0.205) | (0.204) | (0.204) | (0.870) | (0.858) | (0.865) | (0.171) | (0.171) | (0.170) | (0.651) | (0.659) | (0.670) |
| Controls for Electoral margins | 1st Order | 1st Order | 1st Order | 1st Order | 1st Order | 1st Order | 1st Order | 1st Order | 1st Order | 1st Order | 1st Order | 1st Order |
| No individual & district controls | Yes |  |  | Yes |  |  | Yes |  |  | Yes |  |  |
| With Individual controls |  | Yes |  |  | Yes |  |  | Yes |  |  | Yes |  |
| With individual and district controls |  |  | Yes |  |  | Yes |  |  | Yes |  |  | Yes |
| Cohort FE | Yes | Yes | Yes | Yes | Yes | Yes | Yes | Yes | Yes | Yes | Yes | Yes |
| Mother FE | Yes | Yes | Yes | Yes | Yes | Yes | Yes | Yes | Yes | Yes | Yes | Yes |
| **Panel B: 2SLS regressions with 2nd Order polynomials** | | | |  |  |  |  |  |  |  |  |  |
| Proportion of seats won | -0.494** | -0.516** | -0.529*** | 0.914 | 0.818 | 0.600 | -0.246 | -0.260 | -0.279* | 0.650 | 0.617 | 0.534 |
| by female politician | (0.203) | (0.202) | (0.202) | (0.867) | (0.858) | (0.862) | (0.169) | (0.169) | (0.169) | (0.658) | (0.665) | (0.674) |
| Controls for Electoral margins | 2nd Order | 2nd Order | 2nd Order | 2nd Order | 2nd Order | 2nd Order | 2nd Order | 2nd Order | 2nd Order | 2nd Order | 2nd Order | 2nd Order |
| No individual & district controls | Yes |  |  | Yes |  |  | Yes |  |  | Yes |  |  |
| With Individual controls |  | Yes |  |  | Yes |  |  | Yes |  |  | Yes |  |
| With individual and district controls |  |  | Yes |  |  | Yes |  |  | Yes |  |  | Yes |
| Cohort FE | Yes | Yes | Yes | Yes | Yes | Yes | Yes | Yes | Yes | Yes | Yes | Yes |
| Mother FE | Yes | Yes | Yes | Yes | Yes | Yes | Yes | Yes | Yes | Yes | Yes | Yes |
| **Panel C: 2SLS regressions with 3rd Order polynomials** | | | |  |  |  |  |  |  |  |  |  |
| Proportion of seats won | -0.430** | -0.454** | -0.459** | 0.914 | 0.796 | 0.592 | -0.178 | -0.194 | -0.207 | 0.532 | 0.492 | 0.415 |
| by female politician | (0.205) | (0.203) | (0.203) | (1.062) | (1.051) | (1.047) | (0.171) | (0.170) | (0.170) | (0.790) | (0.798) | (0.803) |
| Controls for Electoral margins | 3rd Order | 3rd Order | 3rd Order | 3rd Order | 3rd Order | 3rd Order | 3rd Order | 3rd Order | 3rd Order | 3rd Order | 3rd Order | 3rd Order |
| No individual & district controls | Yes |  |  | Yes |  |  | Yes |  |  | Yes |  |  |
| With Individual controls |  | Yes |  |  | Yes |  |  | Yes |  |  | Yes |  |
| With individual and district controls |  |  | Yes |  |  | Yes |  |  | Yes |  |  | Yes |
| Cohort FE | Yes | Yes | Yes | Yes | Yes | Yes | Yes | Yes | Yes | Yes | Yes | Yes |
| Mother FE | Yes | Yes | Yes | Yes | Yes | Yes | Yes | Yes | Yes | Yes | Yes | Yes |
| Observations | 57,031 | 57,031 | 57,031 | 2,477 | 2,477 | 2,477 | 57,031 | 57,031 | 57,031 | 2,477 | 2,477 | 2,477 |
| Number of mothers | 27,252 | 27,252 | 27,252 | 1,232 | 1,232 | 1,232 | 27,252 | 27,252 | 27,252 | 1,232 | 1,232 | 1,232 |

Note: see note for Table S3.1.

**Table S9:** Robustness checks for results presented in Table 3 by removing outliers in women’s political representation above the 75^th^ percentile.

|  | **(1)** | **(2)** | **(3)** | **(4)** |
| --- | --- | --- | --- | --- |
|  | **Underweight** | | **Severely underweight** | |
|  | **0-60 months** | **0-24 months** | **0-60 months** | **0-24 months** |
| **Panel A: 2SLS regressions with no electoral margins (no polynomials)** |  |  |  |  |
| No individual & district controls: proportion of seats won by female politician | -0.246^**^ | -0.484^**^ | -0.211^**^ | -0.258 |
|  | (0.116) | (0.212) | (0.095) | (0.176) |
| With Individual controls: proportion of seats won by female politician | -0.245^**^ | -0.435^**^ | -0.208^**^ | -0.237 |
|  | (0.115) | (0.209) | (0.095) | (0.176) |
| With individual and district controls: proportion of seats won by female politician | -0.280^**^ | -0.399^*^ | -0.244^***^ | -0.206 |
|  | (0.114) | (0.205) | (0.094) | (0.173) |
| Controls for Electoral margins | No | No | No | No |
| Cohort FE | yes | yes | yes | yes |
| District FE | yes | yes | yes | yes |
| **Panel B: 2SLS regressions with Linear margins** | | | |  |
| No individual & district controls: proportion of seats won by female politician | -0.322^**^ | -0.509^**^ | -0.226^**^ | -0.276 |
|  | (0.137) | (0.240) | (0.112) | (0.198) |
| With Individual controls: proportion of seats won by female politician | -0.323^**^ | -0.507^**^ | -0.224^**^ | -0.283 |
|  | (0.135) | (0.235) | (0.111) | (0.197) |
| With individual and district controls: proportion of seats won by female politician | -0.331^**^ | -0.475^**^ | -0.246^**^ | -0.253 |
|  | (0.134) | (0.235) | (0.111) | (0.198) |
| Controls for Electoral margins | 1st Order | 1st Order | 1st Order | 1st Order |
| Cohort FE | yes | yes | yes | yes |
| District FE | yes | yes | yes | yes |
| **Observations** | **117802** | **44083** | **117802** | **44083** |
| Note: 2rd order & 3rd order polynomials are available on request. These results are similar to the ones presented here.   \| **Table S10: Heterogeneous impacts** \| \| \| \| \| \| --- \| --- \| --- \| --- \| --- \| \|  \| **(1)** \| **(2)** \| **(3)** \| **(4)** \| \|  \| **underweight** \| \| \| \| \|  \| **0-60 months** \| \| **0-24 months** \| \| \| **Subsample of Rural households** \|  \|  \|  \|  \| \| With individual and district controls: proportion of seats won by female politician \| -0.478^***^ \| -0.390^***^ \| -0.434 \| -0.508^*^ \| \| (0.152) \| (0.151) \| (0.277) \| (0.300) \| \| Controls for Electoral margins \| 1st Order \| 3rd Order \| 1st Order \| 3rd Order \| \| Cohort FE \| yes \| yes \| yes \| yes \| \| District FE \| yes \| yes \| yes \| yes \| \| Observations \| *89401* \| \| *33805* \| \| \| **Subsample of Female child** \|  \|  \|  \|  \| \| With individual and district controls: proportion of seats won by female politician \| -0.575^***^ \| -0.414^**^ \| 0.102 \| -0.056 \| \| (0.185) \| (0.182) \| (0.336) \| (0.364) \| \| Controls for Electoral margins \| 1st Order \| 3rd Order \| 1st Order \| 3rd Order \| \| Cohort FE \| yes \| yes \| yes \| yes \| \| District FE \| yes \| yes \| yes \| yes \| \| Observations \| *59152* \| \| *22430* \| \| \| **Subsample of Male child** \|  \|  \|  \|  \| \| With individual and district controls: proportion of seats won by female politician \| -0.421^**^ \| -0.321^*^ \| -0.409 \| -0.336 \| \| (0.177) \| (0.174) \| (0.336) \| (0.374) \| \| Controls for Electoral margins \| 1st Order \| 3rd Order \| 1st Order \| 3rd Order \| \| Cohort FE \| yes \| yes \| yes \| yes \| \| District FE \| yes \| yes \| yes \| yes \| \| Observations \| *63774* \| \| *23919* \| \| \| **Subsample of Household caste: Other backward caste (OBC)** \|  \|  \|  \|  \| \| With individual and district controls: proportion of seats won by female politician \| -0.401^**^ \| -0.345^*^ \| -0.145 \| -0.115 \| \| (0.185) \| (0.197) \| (0.382) \| -0.468 \| \| Controls for Electoral margins \| 1st Order \| 3rd Order \| 1st Order \| 3rd Order \| \| Cohort FE \| yes \| yes \| yes \| yes \| \| District FE \| yes \| yes \| yes \| yes \| \| Observations \| *51675* \| \| *19620* \| \| \| **Subsample of Socially disadvantaged households: SC/ST** \|  \|  \|  \|  \| \| With individual and district controls: proportion of seats won by female politician \| -0.481^**^ \| -0.329 \| -0.144 \| -0.158 \| \| (0.234) \| (0.220) \| (0.405) \| (0.421) \| \| Controls for Electoral margins \| 1st Order \| 3rd Order \| 1st Order \| 3rd Order \| \| Cohort FE \| yes \| yes \| yes \| yes \| \| District FE \| yes \| yes \| yes \| yes \| \| Observations \| *37157* \| \| *14172* \| \| \| **Subsample of households with married spouses** \|  \|  \|  \|  \| \| With individual and district controls: proportion of seats won by female politician \| -0.381^***^ \| -0.264^**^ \| -0.193 \| -0.237 \| \| (0.135) \| (0.133) \| (0.249) \| (0.274) \| \| Controls for Electoral margins \| 1st Order \| 3rd Order \| 1st Order \| 3rd Order \| \| Cohort FE \| yes \| yes \| yes \| yes \| \| District FE \| yes \| yes \| yes \| yes \| \| Observations \| *113993* \| \| *42676* \| \| \| Standard errors in parentheses \|  \|  \|  \|  \| \| ^*^ *p* < 0.1, ^**^ *p* < 0.05, ^***^ *p* < 0.01 \|  \|  \|  \|  \| | | | | |
